# Cost-effectiveness analyses and cost analyses in castration-resistant prostate cancer: A systematic review

**DOI:** 10.1371/journal.pone.0208063

**Published:** 2018-12-05

**Authors:** Thomas Grochtdreis, Hans-Helmut König, Alexander Dobruschkin, Gunhild von Amsberg, Judith Dams

**Affiliations:** 1 Department of Health Economics and Health Services Research, Hamburg Center for Health Economics, University Medical Center Hamburg-Eppendorf, Hamburg, Germany; 2 Department of Oncology, Hematology and Bone Marrow Transplantation with Section Pneumology, Hubertus Wald-Tumorzentrum, University Medical Center Hamburg-Eppendorf, Hamburg, Germany; University of Toronto, CANADA

## Abstract

**Background:**

Treatment of metastatic prostate cancer is associated with high personal and economic burden. Recently, new treatment options for castration-resistant prostate cancer became available with promising survival advantages. However, cost-effectiveness of those new treatment options is sometimes ambiguous or given only under certain circumstances. The aim of this study was to systematically review studies on the cost-effectiveness of treatments and costs of castration-resistant prostate cancer (CRPC) and metastasizing castration-resistant prostate cancer (mCRPC) on their methodological quality and the risk of bias.

**Methods:**

A systematic literature search was performed in the databases PubMed, CINAHL Complete, the Cochrane Library and Web of Science Core Collection for costs-effectiveness analyses, model-based economic evaluations, cost-of-illness analyses and budget impact analyses. Reported costs were inflated to 2015 US$ purchasing power parities. Quality assessment and risk of bias assessment was performed using the Consolidated Health Economic Evaluation Reporting Standards checklist and the Bias in Economic Evaluations checklist, respectively.

**Results:**

In total, 38 articles were identified by the systematic literature search. The methodological quality of the included studies varied widely, and there was considerable risk of bias. The cost-effectiveness treatments for CRPC and mCRPC was assessed with incremental cost-effectiveness ratios ranging from dominance for mitoxantrone to $562,328 per quality-adjusted life year gained for sipuleucel-T compared with prednisone alone. Annual costs for the treatment of castration-resistant prostate cancer ranged from $3,067 to $77,725.

**Conclusion:**

The cost-effectiveness of treatments of CRPC strongly depended on the willingness to pay per quality-adjusted life year gained/life-year saved throughout all included costs-effectiveness analyses and model-based economic evaluations. High-quality cost-effectiveness analyses based on randomized controlled trials are needed in order to make informed decisions on the management of castration-resistant prostate cancer and the resulting financial impact on the healthcare system.

## Introduction

Among men, prostate cancer is the most commonly diagnosed cancer in developed countries and the second most commonly diagnosed cancer worldwide. In 2015, approximately 1.6 million new prostate cancer cases occurred worldwide [1]. Furthermore, prostate cancer was associated with incidence rates of 70 and 15 per 100,000 population and mortality rates of 10 and 7 per 100,000 population in developed countries and developing countries in 2012, respectively [1]. Since 2005, the incidence rate of prostate cancer increased by 66% due to an aging and growing population, and prostate cancer was associated with 6.3 million DALYs globally in 2015 [1]. Besides the increasing disease burden, the economic burden of prostate cancer needs to be considered in particular. For example, in the European Union, prostate cancer has been associated with high total economic costs (€8.4 billion) in 2009, consisting of healthcare costs (€5.4 billion) including medication costs (€3.1 billion), informal care costs (€1.9 billion) and costs due to productivity losses attributable to mortality (€0.7 billion) [2].

It is well known that with increasing incidence rates and rising costs of cancer treatments, a major, even potentially unsustainable, economic burden will affect the governments or public health services [3]. In the past ten years, the number of new effective cancer treatments significantly increased and treatment costs have risen significantly relative to the gross domestic product [4]. Such a significant economic burden might exacerbate in the future, as recently new treatment options for advanced prostate cancer have become available. Therefore, it is pivotal to consider the relationship between additional effects of those new prostate cancer treatments and economic burden to society very carefully [5].

Prostate cancer is mainly treated surgically by radical prostatectomy, hormonally by suppression of endogenous androgens and by definitive radiotherapy [6–9]. However, a considerable amount of prostate cancers become castration resistant/hormone refractory (CRPC; 10–20% within 5 years) and metastasizing (mCRPC; 33% within 2 years of CRPC diagnosis) [10–12]. CRPC is defined as advanced prostate cancer associated with disease progression following surgical or pharmaceutical castration (i.e. continuous rise in serum prostate-specific antigen PSA levels, and/or appearance of new metastases) [11]. Until recently, effective treatment options for CRPC were scarce, however, since 2004, options for treatment of CRPC have impressively evolved [13–19].

For the treatment of patients with non-metastatic CRPC, observation with continued androgen deprivation therapy is currently recommended by guidelines [9, 20–22]. However, apalutamide and enzalutamide recently showed and increased metastasis-free survival and time to symptomatic progression as compared with placebo [23, 24]. For the treatment of patients with asymptomatic or minimally symptomatic mCRPC with good performance status, the androgen receptor targeting therapies abiraterone and enzalutamide [14, 25], chemotherapy with docetaxel [19] or the immunotherapeutic sipuleucel-T [16] are recommended [9, 20, 21, 26, 27]. For patients with symptomatic mCRPC with good performance status, treatment with docetaxel is preferred [19]. For patients with symptomatic mCRPC with poor performance status, treatment with abiraterone, enzalutamide or docetaxel can be considered if feasible. Abiraterone, enzalutamide [13] or chemotherapy with cabazitaxel [15] can be offered to patients with prior docetaxel therapy. For patients with symptomatic metastatic disease limited to the bone, treatment with the radionuclide radium-223 is recommended [17]. Furthermore, bone protective agents should be offered to patients with mCRPC and skeletal metastases to prevent osseous complications. External beam radiotherapy should be considered for localized symptomatic bone metastases [22, 28].

Besides docetaxel, which has proven life-prolonging efficacy for patients with mCRPC, also the therapeutic agents abiraterone, cabazitaxel, enzalutamide, the immunotherapeutic sipuleucel-T and the radionuclide radium-223 showed significant survival advantages [16, 17, 29]. Nevertheless, within the last decade, it has been acknowledged that cost-effectiveness of those therapeutic agents is given only under certain circumstances [30–36]. A recent review examined cost-effectiveness studies in the field of metastatic prostate cancer [37]. The review analyzed 12 studies, focusing on hormonal therapy and 19 studies, focusing on chemotherapy, immunotherapy and medication on cancer-induced bone loss. Single fraction radiotherapy and enzalutamide were mostly considered cost-effective for patients with prior docetaxel treatment, and zoledronic acid was recommended for treatment of patients with symptoms from bone metastases [37]. However, this review did not specifically focus on castration resistant/hormone refractory prostate cancer for which recently new effective, yet high-cost treatment options have become available. Furthermore, this review did not assess the risk of bias specific to economic evaluations. To our knowledge, currently no review systematically examined cost-effectiveness studies or cost studies in the field of CRPC and mCRPC. Therefore, it is important to focus on those new effective, yet high-cost treatment options for CRPC and mCRPC in order to facilitate an informed policy decision making. Therefore, the aim of this study is to systematically review studies on the cost-effectiveness of treatments and costs of CRPC and mCRPC on behalf of their methodological quality and the risk of bias.

## Materials and methods

### Literature search and selection criteria

A systematic literature search was conducted in the databases PubMed, CINAHL Complete, the Cochrane Library (including the National Health Service Economic Evaluations Database, the HTA Database and the Database of Abstracts of Reviews of Effects) and Web of Science Core Collection in September 2017 and was updated in June 2018 to minimize time lag of this review [38]. The following search strategy was used: (neoplasm* OR cancer*) AND (castration resistant OR androgen insensitive OR hormone refractory) AND (prostatic OR prostate) AND (cost* OR economic OR burden* OR marginal analysis OR benefit*). No restrictions were defined according to publication year. Reviews were excluded during eligibility assessment, but screened for further eligible studies. A further manual search for eligible studies has been performed in included studies.

Search results were first independently screened for relevance of title and abstract by two authors (TG and AD). A third author (JD) was involved to reach consensus on disagreement. Second, those articles that were deemed relevant were considered in full text. Articles were excluded if

- they were protocols, letters, editorials, commentaries, conference abstracts, case reports or reviews,
- the study had other objectives than model-based economic evaluations (MEE), cost-effectiveness analyses (CEA), cost-of-illness analyses (COIA) or budget-impact-analyses (BIA) in CRPC and mCRPC, and if
- the full text was not available in English or German.

Full text examination has been conducted independently by two authors (TD and AD). A third author (JD) was involved to reach consensus on disagreement.

### Data extraction and adjustment

Data on study characteristics (e.g. study type, sample size, study perspective, included cost categories), effects, costs, incremental cost-effectiveness ratios (ICERs) and probabilities of cost-effectiveness were extracted and entered into spreadsheets independently by two authors (TG and AD). Included studies were classified as CEA, MEE, COIA or BIA. CEA were defined as economic evaluation using patient-level data, whereas MEE were defined as economic evaluation using decision analytic modelling [39]. Costs were classified into two different perspectives of economic evaluations: the payer’s perspective (e.g. health maintenance organization or NHS) or the societal perspective (costs for productivity losses additionally), according to the consolidated health economic evaluation reporting standards (CHEERS) checklist [40].

Costs reported in the studies were assigned to the following categories: treatment/medication (e.g. chemotherapy, radiation therapy, hormonal therapy, medication administration, health monitoring and adverse events therapy), hospital care (including inpatient care, emergency room visits and treatments), physician and non-physician outpatient care, nursing care, transportation and indirect costs. Furthermore, cost data were inflated to the year 2015 using country-specific gross domestic product inflation rates and converted to international dollars using purchasing power parity (PPP) rates [41]. If no year for the calculation of costs was denoted, the year of study publication was used as base year.

ICERs reported in the studies were generally assigned to either additional cost per quality-adjusted life year (QALY) gained or per life-year saved. Probabilities of cost-effectiveness were defined as the probability that a treatment/medication was cost-effective compared with an alternative treatment/medication or placebo given a maximum acceptable ratio of cost per QALY gained or per life-year saved. A low probability of cost-effectiveness was defined as a probability of around 50% assuming a symmetric distribution of the incremental net benefit of the treatment/medication and the alternative treatment/medication or placebo [42].

### Quality assessment

The methodological quality of CEA/MEE included in this review was assessed using the CHEERS checklist [40, 43]. The CHEERS checklist consists of 24 items arranged in the groups: title and abstract, introduction, methods, results, discussion and other. For the assessment of the methodological quality of COIA, a checklist explicitly developed for the evaluation of COIA has been used [44]. The quality checklist consists of 22 items arranged in the six groups: scope, general economic criteria, calculation of costs, study design and analysis, presentation of results, and discussion. For the assessment of the methodological quality of BIA, currently no valid and reliable instrument exists.

The risk of bias of CEA/MEE was assessed using the Bias in Economic Evaluation (ECOBIAS) checklist [45]. The ECOBIAS checklist consists of 11 items in an overall checklist for bias in economic evaluations and of 11 items in a checklist for model-specific aspects of bias in economic evaluations arranged in three groups: bias related to structure, bias related to data and bias related to consistency. For the assessment of risk of bias of COIA and BIA, currently no valid and reliable instruments exist.

All studies were independently assessed for methodological quality and risk of bias by two authors (TG and JD), and the assessments were compared with each other. Any disagreements were resolved through discussion. The methodical quality and the risk of bias were reported per item. A summary score was not calculated, as weights for different items are non-existent [46]. Thus, no weighting of the studies’ effects, costs, incremental cost-effectiveness ratios and probabilities of cost-effectiveness has been performed.

## Results

### Search results

**Fig 1:**
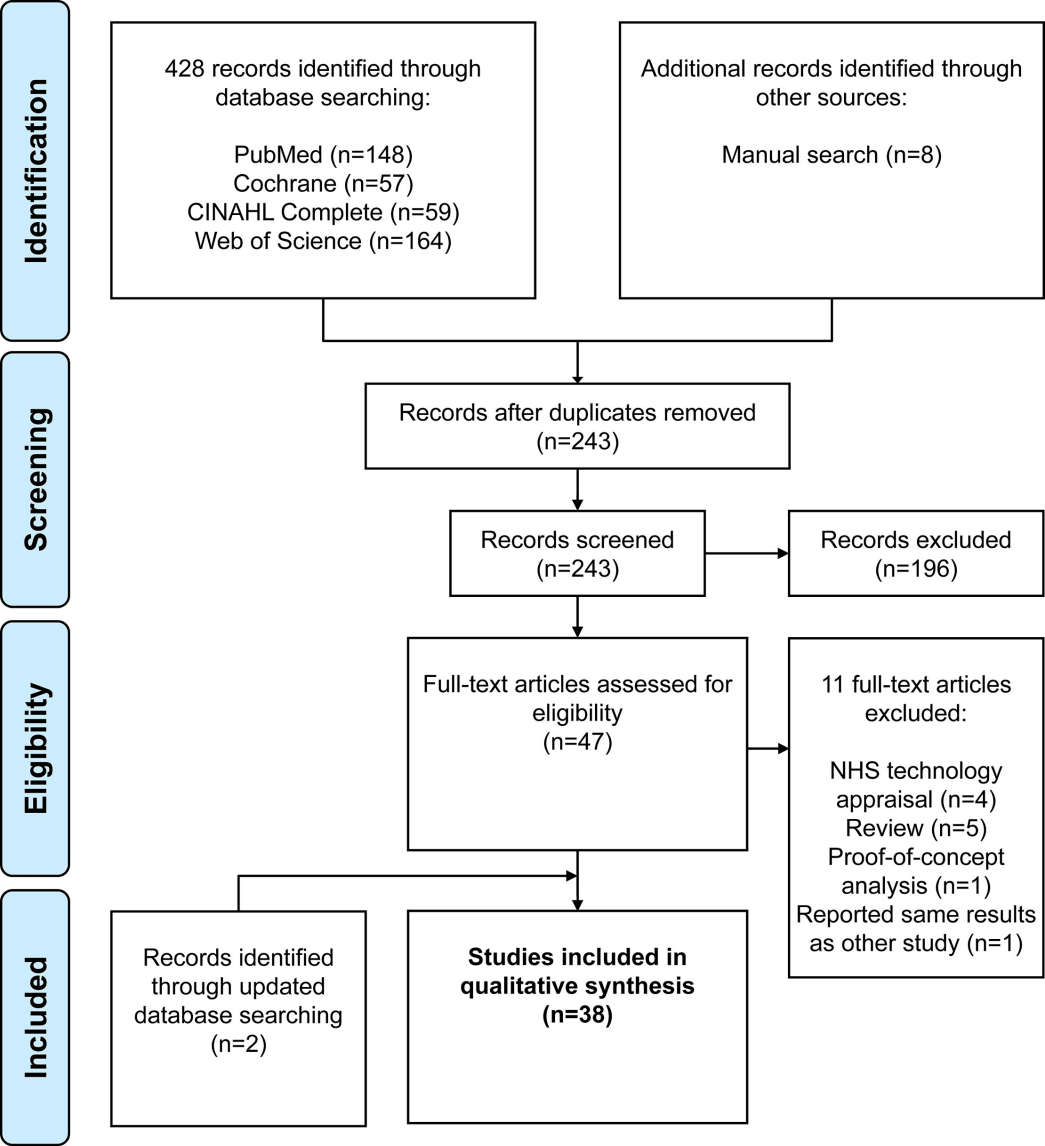
Flow chart of the selection process based on the PRISMA statement [92].

In total, 436 articles were identified by database searching and manual search. Based on title and abstract screening for relevance, 193 duplicates and 196 non-relevant articles were removed (65 protocols, editorials, commentaries or conference abstracts; 120 articles with other study objectives; six articles with full text not available in English or German; and six reviews). A reference list of the excluded non-relevant articles can be found in the S2 File. From the remaining 47 potentially relevant articles, full texts were retrieved and examined for relevance. Based on the full text examination, 11 articles were rejected as they did not meet inclusion criteria (five reviews [47–51]; four technology appraisals of the National Institute for Health and Care Excellence [33–36]; one proof-of-concept-analysis [52]; and one article that reported the same results already found in another study [53]). An update of the systematic literature search identified two [54, 55] additional articles. Finally, 38 articles were included in the review. As one of the articles [56] described both, a MEE and a BIA, this review is based on 39 analyses/evaluations: four CEA [57–60], 15 MEE [54, 56, 61–73], 15 COIA [54, 74–87] and five BIA [56, 88–91]. A flow chart of the selection process is presented in Fig 1.

### Study characteristics

The general characteristics of included CEA/MEE and COIA are presented in Tables 1 and 2, respectively. The general characteristics of included BIA are presented in S1 Table. The majority of the included CEA/MEE originated from the United States (US; n = 12) [62–73], three originated from the United Kingdom (UK) [56, 59, 75], two were multi-country [60, 61], one originated from Canada (CAN) [58] and one from the Netherlands [54], respectively. The included COIA originated majorly from the US (n = 6) [74, 75, 78, 80, 84, 87] and CAN (n = 5) [77, 79, 81, 85, 86] as well as from Japan (n = 2) [55, 82], Ireland (n = 1) [76] or Sweden (n = 1) [83], respectively. The included BIA originated from the US (n = 4) [88–91] and the UK (n = 1) [56], respectively. The earliest study year was 1993 and the most recent study year was 2018.

**Table 1:** General characteristics of included cost-effectiveness analyses and model-based economic evaluations.

Reference	Country	Patients	Sample size (IG,CG)	Mean/median age (IG,CG)	Time horizon	Model type	Data source	Perspective	Year of pricing
***CEA***									
Andronis et al. [57]	UK	mCRPC	707 (350,357)	69^a^	Lifetime	−	RCT	PAY	2012
Bloomfield et al. [58]	CAN	CRCP	114	n.a.	Lifetime	−	RCT	PAY	1996
James et al. [59]	UK	mCRPC	707 (350,357)	69^a^	Lifetime	−	RCT	SOC	2012
Reed et al. [60]	AT, AU, BE, CAN, FR, DE, IT, NZ, SE, CH, UK, US	mCRPC	360 (181,179)	73	15 months	−	RCT	PAY	2000
***MEE***									
Carter et al. [61]	FR, DE, PT, NL	mCRPC	−	72	n.a.	Decision model	Saad et al. [93]	PAY	2007
Collins et al. [56]	UK	mCRPC	−	−	180 months	Markov model	Tannok et al. [19]	PAY	2003
Gong & Hay [62]	US	mCRPC	−	70	Lifetime	Markov model	De Bono et al. [14], Kantoff et al. [16]	SOC	2013
Holko & Kawalec [63]	US	CRPC	−	−	Lifetime	Markov model	Kantoff et al. [16]	PAY	2012
Konski [64]	US	mCRPC with bone metastases	−	−	24 months	Markov model	Various studies [94–100]	PAY	2004^b^
Massoudi et al. [65]	US	mCRPC	−	−	12 months	Statistical analysis	Beer et al. [13], Rathkopf et al. [101]	PAY	2015
Peters et al. [55]	NL	mCRPC	−	−	Lifetime	Markov model	Various studies [15, 17, 18, 102]	SOC	2017
Pilon et al. [66]	US	mCRPC	−	−	n.a.	Statistical analysis	Various studies [13, 25, 101, 103]	PAY	2015
Pollard et al. [67]	US	mCRPC	−	−	Lifetime	Decision-tree model	Various studies [14–19, 104]	PAY	2017^b^
Snedecor et al. [68]	US	mCRPC with bone metastases	−	−	27 months	Markov model	Fizazi et al. [102]	PAY	2010
Stopeck et al. [69]	US	mCRPC with bone metastases	−	−	Lifetime	Markov model	Fizazi et al. [102], Stopeck et al. [105], Henry et al. [106]	PAY	2011
Wilson et al. [70]	US	DX-refractory mCRPC	−	−	18 months	Decision-tree model	De Bono et al. [14], De Bono et al. [15], Scher et al. [18]	PAY	2012
Xie et al. [71]	US	mCRPC with bone metastases	−	−	12 months, 36 months	Markov model	Fizazi et al. [102]	PAY	2010
Zhong et al. [72]	US	DX-refractory mCRPC	−	−	18 months	Decision-tree model	De Bono et al. [14], De Bono et al. [15]	SOC	2010
Zubek & Konski [73]	US	CRPC	−	−	120 months	Markov model	Cordon-Cardo et al. [107]	PAY	2006

AT: Austria, AU: Australia, BE: Belgium, CAN: Canada, CEA: cost-effectiveness analysis, CG: control group, CH: Switzerland, CRPC: castration-resistant prostate cancer, DE: Germany, DX: docetaxel, FR: France, IG: intervention group, IT: Italy, mCRPC: metastatic castration-resistant prostate cancer, MEE: model-based economic evaluation, n.a.: not available, NL: the Netherlands, NZ: New Zealand, PAY: costs are reported from the perspective of a third-party payer, PT: Portugal, SE: Sweden, SOC: costs are reported from the perspective of the society, UK: United Kingdom, US: United States.

^a^ based on a larger data set

^b^ The submission year/study year was assumed as base year.

**Table 2:** General characteristics of included cost-of-illness analyses.

Reference	Country	Patients	Diagnostic criteria/inclusion criteria	Study type	Sample size	Mean age	Data source	Perspective	Year of pricing
Alemayehu et al. [74]	US	CRPC	ICD-9-CM, PSA-level	RCS	349	68	Claims data (commercial, Medicare Advantage)	PAY	2007
		Likely CRPC*	Logistic regression		2,391	74			
Armstrong et al. [75]	US	mCRPC (Medicare 5% sample)	ICD-9	RCS	281	−	Claims data (Medicare, MarketScan commercial)	PAY	2015
		mCRPC (MarketScan dataset)			155				
Bourke et al. [76]	IE	CRPC	−	MA	−	−	Medical literature, study data [108, 109], expert opinion	PAY	2010
Bryant-Lukosius [77]	CAN	CRPC with mental disorder**	TNM, PSA-level, UM-CIDI-SF	PCS	19	69	Self-report (HSUI)	PAY	2001
		CRPC without mental disorder**			80	72			
Bui et al. [78]	US	CRPC with CSS***	ICD-9-CM, PSA-level	RCS	822	74^a^	Claims data (VHA)	PAY	2012
		CRPC without CSS***			177	75^a^			
Dragomir et al. [79]	CAN	mCRPC	−	MA	−	−	Study data [14, 15, 19, 25, 110, 111]	PAY	2013
Engel-Nitz et al. [80]	US	CRPC (oncology cohort)	ICD-9-CM, PSA-level	RCS	1,590	71	Claims data (commercial, Medicare Advantage)	PAY	2008
		CRPC (urology cohort)			995	76			
Krahn et al. [81]	CAN	CRPC	Gleason score, TNM, PSA-level	RCS	46	67^a^	Claims data (Ontario HIP), health care databases^b^	PAY	2008
		mCRPC			46	67^a^			
Kunisawa et al. [82]	JP	Likely CRPC†	Recorded PC diagnoses, docetaxel administration	RCS	13	62^a^	Claims data (Japanese HMO)	PAY	2013
Malmberg et al. [83]	SE	mCRPC‡ (within county)	External radiotherapy use	RCS	46	69	Study data [112], hospital database (UHL)	SOC	1993
		mCRPC‡ (out of county)			33				
Mehra et al. [84]	US	mCRPC	ICD-9, docetaxel administration	RCS	3,642	70	Claims data (IMS LifeLink)	PAY	2012^c^
Organ et al. [85]	CAN	CRPC (intermittent LHRHa)	PSA-level, increase in number or size of metastasis, clinical progression	RCT	18	73	Health administrative databases (Dalhousie University)	PAY	2009
		CRPC (continuous LHRHa)			13	79			
Sanyal et al. [86]	CAN	mCRPC	−	MA	−	−	Study data [113]	PAY	2014
Satoh et al. [55]	JP	mCRPC (ICD-10 sample)	ICD-10, ADT treatment or CRPC-targeted treatment, Japanese MEDIS-DC system	RCS	4,001	72	Claims data (CISA database)	PAY	2016^c^
		mCRPC (MEDIC-DC sample)			276	71			
Sherman et al. [87]	US	mCRPC (strontium)	PSA-level, clinical progression	RCT	7	73	Self-report (COIN form), hospital billing department (MSKCC)	SOC	1997
		mCRPC (CT)			6	66			
		mCRPC (strontium+CT)			7	65			

ADT: androgen deprivation therapy, CAN: Canada, CG: control group, CISA: Clinical Information and Statistical Analysis, COIN: Collection of Indirect and Nonmedical Direct Costs, CRPC: castration-resistant prostate cancer, CSS: corticosteroid-sensitive comorbidities, CT: chemotherapy, HIP: health insurance plan, HMO: health maintenance organization, HSUI: Health Service Utilization Inventory, IE: Ireland, IG: intervention group, JP: Japan, LHRHa: luteinizing hormone-releasing hormone agonists, MA: model approach, mCRPC: metastatic castration-resistant prostate cancer, MEDIS-DC: Medical Information System Development Center, MSKCC: Memorial Sloan-Kettering Cancer Center, PAY: costs are reported from the perspective of a third-party payer, PC: prostate cancer, PCS: prospective cohort study, PSA: prostate-specific antigen, RCS: retrospective cohort study, RCT: randomized controlled trial, SE: Sweden, SOC: costs are reported from the perspective of the society, TNM: TNM Classification of Malignant Tumors, UHL: University Hospital Lund, UM-CIDI-SF: University of Michigan Composite Diagnostic Interview-Short Form, US: United States, VHA: Veterans Health Administration.

* CRPC status was modeled as a function, inter alia, of age, comorbidity, prostate cancer-related costs and docetaxel administration

** outpatient population

*** veteran population

^†^ patients with prostate cancer who had been administered docetaxel were assumed to be CRPC patients

^‡^ patients with bone pain.

^a^ based on a larger data set

^b^ Canadian Institute for Health Information-Discharge Abstract Database, Ontario Drug Benefit Plan database, Complex Continuing Care database, Ontario Home Care Administrative System database, Queen’s University Radiation Oncology Research Unit database

^c^ The submission year/study year was assumed as base year.

The sample size of the CEA and COIA varied from 114 to 707 patients and 13 to 4,001 patients, respectively. The mean/median age varied from 68 to 73 years and 62 to 79 years, respectively. A payer’s perspective was used by three CEA [57, 58, 60], 11 MEE [56, 61, 63–69, 71, 73] and 13 COIA [55, 74–82, 84–86]. A societal perspective was used by one CEA [59], four MEE [54, 62, 70, 72] and two COIA [83, 87]. Ten CEA/MEE reported a lifetime follow-up [54, 56–59, 62, 63, 66, 67, 69]. Nine CEA/MEE reported varying lengths of follow-up from 12 to 120 months. The COIA were based majorly on retrospective cohort studies (n = 9) [55, 74, 75, 78, 80–84]. Three COIA used a model approach [76, 79, 86], two were based on randomized controlled trials [85, 87], and one was based on a prospective cohort study [77]. A length of follow-up varying per patient was reported by seven COIA [74, 78, 80–84], whereas the remaining COIA reported a length of follow-up from six to 24 months [55, 75–77, 85, 87] or a lifetime follow-up [79, 86].

### Methodological quality and risk of bias

The CEA/MEE fulfilled 43% to 95% of the CHEERS-criteria [40] (S2 and S3 Tables). Only 26% of the CEA/MEE stated relevant aspects of the study setting [56, 58, 61, 62, 71] and only 47% of the MEE described fully the methods used for identification of included studies and synthesis of clinical effectiveness data [54, 56, 61–64, 72]. The structure of the decision analytic model used for analysis and its appropriateness for use in the study was adequately described by 40% of all MEE [54, 62, 68, 69, 71, 73].

The COIA fulfilled 61% to 90% of the criteria methodological quality checklist for COIA [44] (S4 Table). Only 47% of the studies identified eligible patients based on objective diagnostic criteria [55, 74, 75, 78, 80, 81, 84] and only 27% of the studies included the costs restricted to those definitely attributable to CRPC [79, 83, 86]. Furthermore, only 17% of the applicable studies discounted future costs [83, 86] and only 20% conducted univariate and/or probabilistic sensitivity analyses [79, 82, 86].

The CEA/MEE fulfilled 19% to 100% of the ECOBIAS-criteria [45] (S5 and S6 Tables). Only one CEA considered uncertainty in sufficient detail in a sensitivity analysis, disclosed sponsorships, listed the study in a trial register and reported results according to a freely accessible study protocol [59].

### Cost-effectiveness analyses

One analysis calculated the cost-effectiveness of mitoxantrone compared with prednisone alone for patients with CRPC based on a randomized controlled trial [58]. Mitoxantrone was dominant (i.e. less costly and more effective) compared with prednisone alone [58].

Five analyses calculated the cost-effectiveness of the bone-targeted therapies zoledronic acid compared with no zoledronic acid [57, 59] and compared with placebo [60], as well as strontium-89 compared with no strontium-89 [57, 59] for patients with mCRPC. The ICER of zoledronic acid compared with no zoledronic ranged between $11,468 and $42,047 per QALY gained [57, 59]. The ICERs of zoledronic acid compared with placebo was $213,513 per QALY gained or $16,496 per skeletal-related event avoided, respectively [60]. The ICER of strontium-89 compared with no strontium-89 ranged between $16,590 and $24,187 per QALY gained [57, 59].

### Model-based economic evaluations

The cost-effectiveness of abiraterone compared with placebo/prednisone alone for patients with mCRPC was modeled by three analyses with ICERs of $112,100 per life-month saved [66] and of $128,895 [70] to $399,525 [62] per QALY gained. One further analysis indicated an ICER of abiraterone compared with mitoxantrone of $98,939 per QALY gained [72]. The health effects, costs, ICERs and probabilities of cost-effectiveness of all MEE are presented in Table 3.

**Table 3:** Cost-effectiveness analyses and model economic evaluations–health effects, costs, cost-effectiveness ratios and probabilities of cost-effectiveness.

Reference	Comparator	Cost categories^a^	Incremental health effects	Incremental costs (in $-PPP)	ICER (in $-PPP per additional health effect)	Probability of cost-effectiveness (per additional health effect)
***CEA***						
Andronis et al. [57]	With ZA vs. without ZA	A, B, C	0.03 QALYs gained	360	11,468	64% for a WTP of $42,976
	With S89 vs. without S89		0.08 QALYs gained	1,955	24,187	60% for a WTP of $42,976
Bloomfield et al. [58]	M+P vs. P	A, B, C	0.26 QALYs gained	−2,051	Dominant	−
James et al. [59]	With ZA vs. without ZA	A, B, C	0.03 QALYs gained	1,319	42,047	40% for a WTP of $42,976
	With S89 vs. without S89		0.08 QALYs gained	1,341	16,590	76% for a WTP of $42,976
Reed et al. [60]	ZA vs. placebo	A, B, C, D	0.46 SRE avoided	−435^b^	16,496^c^	−
			0.04 QALYs gained		213,513^c^	
***MEE***						
Carter et al. [61]	ZA vs. placebo	A	0.04 QALYs gained	1,741 (FR)	48,833	−
				1,182 (DE)	31,136	
				377 (PT)	15,605	
				116 (NL)	3,283	
Collins et al. [56]– 1^st^ analysis	P vs. M+P	A	−0.00 QALYs gained	737	Dominated	26% for a WTP of $74,965
	DX+P vs. M+P		0.15 QALYs gained	9,462	70,666	53% for a WTP of $74,965
Collins et al. [56]– 2^nd^ analysis	M+P+C vs. M+P	A	−0.02 QALYs gained	326	Dominated	12% for a WTP of $74,965
	P vs. M+P		−0.00 QALYs gained	737	Dominated	16% for a WTP of $74,965
	DX+P(weekly) vs. DX+P		−0.12 QALYs gained	28,925	Dominated	0% for a WTP of $74,965
	DX70*+ES+P vs. DX+P		−0.10 QALYs gained	10,169	Dominated	16% for a WTP of $74,965
	DX35**+ES+P vs. DX+P		−0.07 QALYs gained	14,292	Dominated	4% for a WTP of $74,965
	DX+E vs. DX+P		0.13 QALYs gained	7,875	ED	25% for a WTP of $74,965
	DX+P(3-weekly) vs. M+P		0.15 QALYs gained	9,462	61,295	20% for a WTP of $74,965
Gong & Hay [62]	A vs. P	A, B, C	0.43 QALYs gained	174,670	399,525	50% for a WTP of $410,985
	ST vs. P		0.16 QALYs gained	93,921	562,328	50% for a WTP of $277,415
Holko et al. [63]	ST vs. SC	A, B, C	0.37 QALYs gained	109,164	295,529	4% for a WTP of $155,597
Konski [64]	SFX RT vs. Rx	A	0.03 QALYs gained	247	8,454	−
	MFX RT vs. Rx		0.04 QALYs gained	1,849	44,386	
	M+P vs. Rx		−0.07 QALYs gained	4,439	Dominated	
Massoudi et al. [65]	EZ vs. A+P	A, B, D	NNT 14 (free of progression or death)	−2,666	Dominant	−
			NNT 26 (CT delayed)		Dominant	
			NNT 91 (death avoided)		Dominant	
Peters et al. [55]	R223 vs. A	A, B, C, D, E	0.02 QALYs gained	−7,475^d^	Dominant	61% for a WTP of 98,160^d^
	R223 vs. CX		0.01 QALYs gained	−5,479^d^	Dominant	54% for a WTP of 98,160^d^
	R223 vs. EX		−0.06 QALYs gained	−9,067^d^	Less costly/effective	61% for a WTP of 98,160^d^
Pilon et al. [66]	A+P vs. placebo+P	A	4.40 LMS	112,100^d^	3,231^d^	−
	EZ vs. placebo		4.00 LMS	159,264^d^	4,512^d^	
Pollard et al. [67]– 1^st^ analysis	ST vs. SC	A	0.34 LYS	106,117^d^	312,109^d^ (ED)	−
	ST+EZ^e^		0.31 LYS	68,384^d^	220,594^d^ (ED)	
	ST+EZ+A^e^		0.33 LYS	50,119^d^	151,876^d^ (ED)	
	ST+EZ+A+DX vs. SC		1.18 LYS	245,103^d^	207,714^d^	
	ST+EZ+A+DX+R223^e^		0.30 LYS	80,072^d^	266,907^d^	
	ST+EZ+A+DX+R223+CX^e^		0.20 LYS	54,287^d^	271,435^d^	
Pollard et al. [67]– 2^nd^ analysis	EZ vs. SC	A	0.31 LYS	68,384	220,594 (ED)	−
	EZ+A^e^		0.33 LYS	50,119	151,876 (ED)	
	EZ+A+DX vs. SC		0.84 LYS	138,986	165,460	
	EZ+A+DX+R223^e^		0.30 LYS	80,072	266,907	
	EZ+A+DX+R223+CX^e^		0.20 LYS	54,287	271,435	
Snedecor et al. [68]	D vs. ZA	A,B,C	0.01 QALYs gained	8,507	1,148,734	0% for a WTP of $108,500
Stopeck et al. [69]	D vs. ZA	A,B,C	0.81 SRE avoided	7,343	9,104	−
			0.14 QALYs gained		52,502	83% for a WTP of $106,268
Wilson et al. [70]	A+P vs. placebo (P)	A+B	0.27 QALYs gained	35,265	128,895	29% for a WTP of $104,427
	EZ+P vs. A+P		0.03 QALYs gained	13,648	456,998	21% for a WTP of $104,427
	CX+P vs. EZ+P		0.06 QALYs gained	21,177	367,443	16% for a WTP of $104,427
Xie et al. [71]	D vs. ZA (12 months)	A	0.11 SRE avoided	8,477	77,064	17.5% for a WTP of $54,250
	D vs. ZA (36 months)		0.27 SRE avoided	15,034	55,681	49.8% for a WTP of $54,250
Zhong et al. [72]	M vs. placebo	A+B	0.08 QALYs gained	8,468	109,232	−
	A vs. M		0.20 QALYs gained	19,400	98,939	42% for a WTP of 108,500$
	CX vs. A		0.06 QALYs gained	59,772	1,037,111	5% for a WTP of 108,500$
Zubek & Konski [73]	PPT vs. SC	A	1.64 QALYs gained	3,989	2,432	100% for a WTP of $57,898
	KN vs. SC		0.92 QALYs gained	67	73	90% for a WTP of $57,898
	PPT vs. KN		0.72 QALYs gained	3,922	5,447	100% for a WTP of $57,898

A: abiraterone, C: clondrate, CT: chemotherapy, CX: cabazitaxel, D: denosumab, DE: Germany, DX: docetaxel, ES: estramustine, ED: extended dominance, EZ: enzalutamide, FR: France, FU: follow-up, KN: Kattan nomogram, LMS: life-months saved, LYS: live-years saved, M: mitoxantrone, MFX: multiple fractions of external beam radiotherapy, NL: the Netherlands, NNT: number needed to treat, P: prednisone/prednisolone, PPT: prostate Px test, PT: Portugal, QALY: quality-adjusted life year, R223: radium-223, Rx: pain medication, S89: strontium-89, SC: standard care, SFX: single fraction of external beam radiotherapy, SRE: skeletal-related events, ST: sipuleucel-T, WTP: willingness to pay, ZA: zoledronic acid.

* 75mg/m^2^

** 30mg/m^2^

^a^ Costs reported in the studies were assigned to the following categories: (A) treatment, (B) hospital care, (C) physician and non-physician outpatient care, (D) nursing care, (E) productivity loss

^b^ Excluding treatment costs

^c^ Including only treatment costs

^d^ In $-PPP-2017 without inflation

^e^ versus prior analyzed intervention.

The cost-effectiveness of docetaxel for patients with mCRPC was modeled by two analyses of Collins et al. [56] with an ICER of $70,666 per QALY gained for 75 mg/m^2^ docetaxel every 3 weeks compared with mitoxantrone. In the second analysis, the treatment regimen containing 60–70 mg/m^2^ docetaxel every 3 weeks was extendedly dominant, i.e. an ICER higher than that of the next most effective regimen, compared with all other treatment regimen (i.e. combinations containing mitoxantrone, docetaxel or estramustine) [56].

The cost-effectiveness of Sipuleucel-T for patients with CRPC [63] and mCRPC [62, 67] was modeled in three analyses. Two analyses indicated ICERs of $295,538 [63] to $562,328 [62] per QALY gained for Sipuleucel-T compared with standard care/prednisone alone. The third analysis indicated extended dominance of drug regimens containing Sipuleucel-T, enzalutamide and abiraterone as well as an ICER of $207,714 per life-year saved for a drug regimen containing additionally docetaxel [67].

Extended dominance was also indicated for drug regimens containing enzalutamide and abiraterone and the ICER for a drug regimen containing additionally docetaxel was $165,460 per life-year saved [67]. Three other analyses modeled the cost-effectiveness of enzalutamide for patients with mCRPC [65, 66, 70]. Two analyses reported an ICER of $456,998 per QALY gained compared with abiraterone [70] as well as dominance of enzalutamide [65], respectively. The third analysis indicated an ICER of $4,512 per life-month saved compared with placebo [66].

One analysis modeled the cost-effectiveness of single and multiple fraction external beam radiotherapy for patients with mCRPC with bone metastases compared with supportive care with ICERs of $8,454 and $44,386 per QALY gained, respectively [64]. The models of cost-effectiveness analyses of mitoxantrone [56, 64, 72], cabazitaxel [70, 72], radium-223 [54] and denosumab [68, 69, 71] for patients with mCRPC are presented exclusively in Table 3. Furthermore, the model of a cost-effectiveness analysis of risk-prediction tools in selecting patients for immediate post-prostatectomy treatment [73] are also presented exclusively in Table 3.

### Cost-of-illness analyses

The overall annual direct costs of patients with CRPC and mCRPC ranged from $2,474 [85] to $50,537 [74] and from $26,707 [87] to $67,957 [75], respectively. The annual cancer-specific costs of patients with CRPC and mCRPC ranged from $14,335 [80] to $77,725 [76] and from $3,067 [86] to $73,270 [84], respectively. The costs for hospital care and outpatient care ranged from $611 [55] to $23,308 [84] per year and $507 [83] to $41,170 [80] per year, respectively. The medication costs and the costs for nursing care ranged from $3,425 [77] to $36,864 [75] per year and $490 [75] to $1,475 [81] per year, respectively. No COIA included indirect costs (Table 4).

**Table 4:** Cost-of-illness analyses–Costs in categories and total costs per patient per year (in 2015 US$-PPP).

Reference	Time horizon	Hospital care cost^a^	Outpatient care cost	Medication costs	Nursing care costs	Overall direct costs^b^	CRCP-specific costs
Alemayehu et al. [74]	Varying1	17,121	25,052	3,993	−	47,465	24,348
Known CRPC		15,835	28,598	4,683	−	50,537	30,412
Likely CRPC		17,324	24,538	3,898	−	47,018	23,468
Armstrong et al. [75]	24 months						
Medicare		7,362	6,927	12,802	1,248	28,792	−
MarketScan		10,109	19,827	36,864	490	67,957	−
Bourke et al. [76]	12 months	−	−	−	−	−	77,725
Bryant-Lukosius [77]	12 months	1,698	1,840	3,425	550	7,514	−
Bui et al. [78]	Varying^1^						
With CSS		4,286	29,323	24,962	−	59,799	−
W/o CSS		14,499	31,604	24,148	−	71,742	−
Dragomir et al. [79]	Lifetime						
ADT+DX+A treatment		−	−	−	−	−	17,441
ADT+DX+CX treatment		−	−	−	−	−	28,242
Engel-Nitz et al. [80]	Varying^2^						
Oncologist patient		22,364	41,170	5,314	−	70,627	47,647
Urologist patient		17,670	11,240	3,578	−	33,918	14,335
Krahn et al. [81]	Varying^3^						
CRPC		14,543	4,491	6,022	433	25,490	−
mCRPC		19,243	5,482	9,810	1,475	36,010	−
Kunisawa et al. [82]	Varying^1^	−	−	−	−	17,921	−
Malmberg et al. [83]	Varying^4^						
Within country		3,743	520	−	−	−	4,996
Out of country		6,725	507	−	−	−	7,827
Mehra et al. [84]	Varying^1^						
Pre-DX period		8,960	18,408	5,125	−	−	32,494
Post-DX period		23,308	25,614	24,323	−	−	73,270
Organ et al. [85]	24 months						
Intermittent LHRHa		−	−	−	−	2,474	−
Continuous LHRHa		−	−	−	−	6,514	−
Sanyal et al. [86]	Lifetime	−	−	−	−	−	3,067
Satoh et al. [55]	Varying^5^						
ICD-10		611^c^	−	6,183^c^	−	−	6,794^c^
MEDIC-DC		1,183^c^	−	19,935^c^	−	−	21,118^c^
Sherman et al. [87]	6 months	−	−	−	−	31,683	−
S89 treatment		−	−	−	−	26,707	−
V+E treatment		−	−	−	−	28,216	−
V+E+S89 treatment		−	−	−	−	51,162	−

ADT: androgen deprivation therapy, CRCP: castration-resistant prostate cancer, CSS: corticosteroid-sensitive comorbidities, CX: cabazitaxel, DX: docetaxel, E: estramustine LHRHa: luteinizing hormone-releasing hormone agonist, mCRPC: metastatic castration-resistant prostate cancer, S89: strontium-89, V: vinblastine, w/o: without.

^1^ Costs per patient per month were reported

^2^ costs per patient per 6 months were reported

^3^ costs per patient per 100 days were reported

^4^ costs per patient per relapse were reported

^5^ costs per patient per 12 months were reported

^a^ including costs for emergency department visits

^b^ may include other medical, non-medical or indirect costs not previously listed

^c^ in $-PPP-2016 without inflation.

### Budget-impact analyses

The yearly budget-impact of adopting docetaxel for patients with mCRPC on the UK healthcare system was estimated to be $356,580 to $386,986 per million population [56]. The yearly budget-impact of adopting enzalutamide for patients with mCRPC on the US healthcare system was indicated to be $515,871 per million population [88]. The yearly budget-impact of adopting cabazitaxel and abiraterone for docetaxel-refractory patients with mCRPC on the US healthcare system was indicated to be $6,331,704 [91] and $36,035 to $105,982 [90] per million population, respectively. The adoption of a test for the prediction of non-response to the hormonal therapies abiraterone and enzalutamide for patients with mCRPC (AR-V7 testing) in the US healthcare system was associated with yearly cost savings of $468,854 per million population [89] (S1 Table).

## Discussion

The aim of this study was to systematically review studies on the cost-effectiveness of treatments and costs of CRPC and mCRPC on behalf of their methodological quality and the risk of bias. In total, 19 CEA/MEE and 20 COIA/BIA were identified and included.

It is noteworthy that no article reported a COIA investigating the excess costs of CRPC and mCRPC using an econometric approach for cost estimation [114]. Furthermore, COIA that were included in this systematic review only reported either treatment related costs or partial costs from a narrow perspective. Notably, only COIA investigating the excess costs of a disease from a societal perspective should be the basis for health care prioritization discussions. For the allocation of health care resources, economic evaluations play an important role [39]. MEE bring together the evidence of a range of real-life sources, such as CEA based on randomized controlled trials, and provide a framework for decision-making. However, if the evidence for MEE to be based on is scarce or even inaccurate, bias might be induced to the ICER and, as a consequence, lead to invalid health care resource allocation-decisions [45]. Therefore, it is pivotal to determine uncertainty of the cost-effectiveness of treatments by means of sensitivity analyses and to conduct CEA based on randomized controlled trials as basis for future MEE.

### Cost-of-illness analyses and budget-impact analyses

Annual direct healthcare costs of patients with mCRPC were relatively high (up to $68,000) and budget-impacts of adopting new treatments for patients with CRPC and mCRPC to healthcare systems (e.g. up to $387,000 per million population per year for the adoption of docetaxel) appear to be relevant. Therefore, conducting CEA and MEE with a high methodological quality and a low risk of bias are advisable for adequacy of reimbursement decisions. Furthermore, costs for outpatient care and nursing care should be regularly included to the calculation of cost in CEA and MEE of treatments of CRPC and mCRPC. The current review showed that annual costs of care were as high as costs for medication or hospital care. However, it has to be acknowledged that costs of cancer medication still have the greatest impact on the ICER of CRPC and mCRPC treatment [37].

### Cost-effectiveness analyses and model-based economic evaluations

Currently, there is no generally accepted willingness to pay (WTP) threshold per QALY gained. In the UK, a cost-effectiveness threshold ranging between £20,000 ($30,000) and £30,000 ($40,000) per QALY gained has been defined [115, 116]. For treatments extending life at the end of life, a threshold of £50,000 ($70,000) per QALY gained has been defined [117, 118]. In the US, cost-effectiveness thresholds usually range between $50,000 and $100,000 per QALY gained [119–121]. Notwithstanding, a review on WTP thresholds for oncology drugs reported that those were regularly in the range of $100,000 to $150,000 per QALY gained [122].

For abiraterone and enzalutamide, CEA/MEE showed unfavorable ICERs above or around a regularly used WTP threshold for oncology drugs of $100,000 per QALY gained with a low probability of cost-effectiveness [122]. In the UK and the US, both, abiraterone and enzalutamide are recommended for treatment of mCRPC for patients with no prior docetaxel treatment [31, 32]. Sipuleucel-T is only recommended in the US for treatment of asymptomatic or minimally symptomatic mCRPC for patients with no prior docetaxel treatment [123, 124]. The current systematic review yielded ICERs way above a WTP threshold of $100,000 per QALY gained for sipuleucel-T compared with standard care with low probabilities of cost-effectiveness.

Docetaxel showed an ICER below a WTP threshold of $100,000 per QALY gained in one MEE. However, the probability of cost-effectiveness was only around 50% for a WTP of around $75,000 per QALY gained. In the UK and the US, docetaxel is recommended for treatment of mCRPC [33, 125]. For patients with docetaxel-refractory mCRPC, cabazitaxel, enzalutamide and abiraterone plus prednisone can be considered for treatment, yet in the UK only under certain circumstances (i.e. manufacturer discount) [34–36, 124, 126, 127]. All three treatments showed unfavorable ICERs above or around a WTP of $100,000 per QALY gained with low probabilities of cost-effectiveness for patients with docetaxel-refractory mCRPC in the current review.

One comparative MEE showed that radium-223 was dominant compared with abiraterone and cabazitaxel for patients with mCRPC [55]. Yet, the probabilities of cost-effectiveness were only 61% and 54% for a WTP of around $100,000 per QALY gained. Two further MEE showed unfavorable ICERs even above a WTP of $100,000 per life-year saved for treatment regimen containing radium-223 compared with treatment regimen without radium-223 [67]. Nevertheless, it must be mentioned that the latter MEE fulfilled only 43% of the CHEERS-criteria, indicating a lower methodological quality. However, radium-223 is recommended for treatment of docetaxel-refractory mCRPC with bone metastases in both, the UK and US [30, 128]. Denosumab is only recommended in the US for the prevention of skeletal-related events in patients with bone metastases from solid tumors [129, 130]. The current review yielded divergent ICERs comparing denosumab with zoledronic acid that were either way above or even around $50,000 per QALY gained with probabilities of cost-effectiveness ranging from 0% to 83% for a WTP of around $100,000 per QALY gained.

Two recently published phase 3 studies have shown favorable results of enzalutamide and apalutamide for patients with non-metastatic CRPC concerning metastasis-free survival and time to symptomatic progression compared with placebo [23, 24]. However, the cost-effectiveness as well as the budget-impact of both enzalutamide and apalutamide for patients with non-metastatic CRPC has not yet been evaluated.

### Methodological quality and risk of bias

Although the ICER has a high priority in health economics and the decision making in the context of technology appraisals, methodological quality and risk of bias of cost-effectiveness analyses play an important role in the credibility of this outcome. The methodological quality of the included CEA and MEE in this systematic review ranged widely from 43% to 95% of CHEERS-criteria fulfilled. Particularly the choice of model in MEE was accountable for a reduced methodological quality. Markov chain models should be preferred for modelling the cost-effectiveness of mCRPC, as it is possible to model longer periods with uncertain timing of death [39, 131, 132]. Current treatment options for mCRPC have shown to prolong survival in patients, whereby the risk of adverse events related to their treatment is high [133, 134]. Consequently, decision tree models are inadequate to capture complex scenarios, as in current treatment options for mCRPC [131, 132]. Nevertheless, 40% of all MEE of the current review did actually not use adequate models, such as Markov chain models, for modelling the cost-effectiveness of mCRPC and therefore, presumably introduced bias to the evaluation [45]. The ECOBIAS-criteria for risk of bias fulfilled by CEA/MEE ranged widely between 19% and 100%. It is worth mentioning that uncertainty of the cost-effectiveness of treatments was considered in sufficient detail in sensitivity analyses by merely one CEA. Moreover, more than two thirds of MEE did not make the methods used to identify data transparent and therefore might have induced bias to the results [45].

The methodological quality of the included COIA was relatively high, as 61% to 90% of the criteria from the checklist explicitly developed for the evaluation of COIA from Stuhldreher et al. [44] were fulfilled. However, as already mentioned, no study included a non-diseased comparison group to the analysis, yet about one third of the COIA reported only disease-specific costs. Furthermore, methodological quality was impaired by misleading or missing information about discounting of costs. Moreover, relevant parameters were not varied in sensitivity analyses in order to test the robustness of the results in the vast majority of COIA in the current review [44].

### Strengths and limitations

To our knowledge, this is the first systematic review of costs and cost-effectiveness in CRPC and mCRPC. Compared with another systematic review that examined cost-effectiveness studies in the field of metastatic prostate cancer [37], we additionally included studies in the field of CRPC, where effective treatment options have impressively evolved in recent years. Moreover, the focus of the current systematic review was on economic aspects of CRPC and mCRPC. Therefore, we structured the systematic review by type of economic analysis rather than by treatment option alone to facilitate comparability across treatment options. Furthermore, we additionally analyzed the risk of bias of the included studies. This review was the first to bring together the evidence of MEE, CEA, COIA and BIA in CRPC and mCRPC comprehensively and systematically. The methodological quality and risk of bias of included studies were analyzed and described using the current checklists CHEERS, ECOBIAS and the COIA checklist from Stuhldreher et al. [40, 44, 45]. Furthermore, we were able to improve comparability and interpretability of the studies, as we inflated cost data to the year 2015 and converted cost data to international dollars. However, comparability and interpretability of the studies is still limited, as they differed in characteristics of included patients (e.g. diagnostic criteria/inclusion criteria and age), study type, sample size or study perspective and methodological quality and risk of bias across studies was heterogeneous.

As we did not include grey literature in this systematic review, publications were probably omitted and, therefore, a potential publication bias was introduced. Subsequently, we also decided against inclusion of technology appraisals for the government or public health services (e.g. [33–36]), as the analysis of the methodological quality and risk of bias as well as the data extraction and adjustment would have been limited to the published information, as the corresponding manufacturer submissions were not available. Generalizability of the results of the systematic review was limited to the health care system of the respective study, as health care utilization and the resulting costs across health care systems are difficult to compare. Furthermore, external validity of the results of the systematic review was limited due to heterogeneous characteristics of the included studies (i.e. varying time horizons, sample sizes, study perspectives, included cost categories). Due do heterogeneity of the included studies, a formal meta-analysis was not performed, rendering the systematic review narrative in nature. However, accuracy and usability of the findings of this review were enhanced by inflation of cost data and conversion to international dollars. Lastly, studies with insignificant results might have been omitted due to publication bias.

## Conclusions

Existing CEA/MEE included in this systematic review produced a very heterogeneous picture regarding the value for money for different recommended chemotherapies, androgen receptor targeting therapies, immunotherapeutic agents and radionuclides for the treatments of CRPC and mCRPC. Furthermore, the improvable methodological quality and a relatively high risk of bias of CEA/MEE introduce great uncertainty to the overall interpretation of the study results. There is a great need for high-quality CEA based on randomized controlled trials in order to determine cost-effectiveness of CRPC and mCRPC treatments, to bring together this evidence in MEE and finally to protect patients from unfavorable health outcomes and the society from unjustified healthcare system costs.

## Supporting information

S1 Table Budget-impact analyses–General characteristics, incremental costs per member per month and incremental total costs (in 2015 US$-PPP). A: abiraterone, AR-V7: Androgen receptor variant-7, CX: cabazitaxel, DX: docetaxel, EZ: enzalutamide, mCRPC: metastatic castration-resistant prostate cancer, P: prednisone/prednisolone, PAY: payer’s perspective UK: United Kingdom, US: United States. * Based on United Nations estimates of the UK population in 2015 (65,397,080) [133], ** based on United Nations estimates of the US population in 2015 (319,929,162) [133], *** the submission year/study year was assumed as base year.
(PDF)

S2 Table Quality assessment of included cost-effectiveness analyses (based on the CHEERS-checklist [40]). ✓: Criterion fulfilled; CHEERS: consolidated health economic evaluation reporting standards, n.a.: not applicable.
(PDF)

S3 Table Quality assessment of included model-based economic evaluations (based on the CHEERS-checklist [40]). ✓: Criterion fulfilled, CHEERS: consolidated health economic evaluation reporting standards, n.a.: not applicable.
(PDF)

S4 Table Quality assessment of included cost-of-illness analyses (based on Stuhldreher et al. [43]). ✓: Criterion fulfilled, n.a.: not applicable.
(PDF)

S5 Table Risk of bias assessment of included cost-effectiveness analyses (based on the ECOBIAS checklist [44]). ✓: Criterion fulfilled, (✓): criterion partially fulfilled, ECOBIAS: Bias in Economic Evaluation, n.a.: not applicable.
(PDF)

S6 Table Risk of bias assessment of included model-based economic evaluations (based on the ECOBIAS checklist [44]). ✓: Criterion fulfilled, (✓): criterion partially fulfilled, ECOBIAS: Bias in Economic Evaluation, n.a.: not applicable. * These biases are overlapping regarding their content, ** these biases are overlapping regarding their content.
(PDF)

S1 File PRISMA 2009 checklist. (PDF)

S2 File References of the excluded non-relevant articles. (PDF)
