# Supplementary material for: Cost-effectiveness analyses and cost analyses in castration-resistant prostate cancer: A systematic review
Source: PLoS One. 2018 Dec 5;13(12):e0208063. doi: 10.1371/journal.pone.0208063 (PMC6281264; doi:10.1371/journal.pone.0208063)
Supplement: S1 Table — A: abiraterone, AR-V7: Androgen receptor variant-7, CX: cabazitaxel, DX: docetaxel, EZ: enzalutamide, mCRPC: metastatic castration-resistant prostate cancer, P: prednisone/prednisolone, PAY: payer’s perspective UK: United Kingdom, US: United States. * Based on United Nations estimates of the UK population in 2015 (65,397,080) [133], ** based on United Nations estimates of the US population in 2015 (319,929,162) [133], *** the submission year/study year was assumed as base year. (PDF) [file pone.0208063.s001.pdf]

S1 Table. Budget-impact analyses – General characteristics, incremental costs per member per month and incremental total costs (in 2015 US$-PPP)

Reference	Country	Patients	Eligible population (per 1,000,000 per year)	Intervention	Perspective	Year of pricing	Time horizon (months)	Cost of new intervention (per month)	Incremental costs per member (per month)	Incremental total costs (per 1,000,000 per year)
Bui et al. [88]	US	mCRPC	115	EZ	PAY	2014	12	8,604	0.040	515,871
Collins et al. [56]	UK	mCRPC	42*	DX+P	PAY	2004	12	8,487-9,206	–	356,580-386,968*
Flannery et al. [91]	US	DX-refractory mCRPC	12	CX	PAY	2015	12	5,054	0.528	6,331,704
Markowski et al. [89]	US	mCRPC	94**	AR-V7 testing	PAY	2016***	12	1,000	–	−468,854**
Sorensen et al. [90]	US	DX-refractory mCRPC	5.7	A+P	PAY	2012	18	6,075	0.009-0.012	36,035-105,982

A: abiraterone, AR-V7: Androgen receptor variant-7, CX: cabazitaxel, DX: docetaxel, EZ: enzalutamide, mCRPC: metastatic castration-resistant prostate cancer, P: prednisone/prednisolone, PAY: payer's perspective UK: United Kingdom, US: United States.

* Based on United Nations estimates of the UK population in 2015 (65,397,080) [135], ** based on United Nations estimates of the US population in 2015 (319,929,162) [135], *** the submission year/study year was assumed as base year.

References

135. United Nations DoEaSA, Population Division,. World population prospects: the 2017 revision, DVD edition; 2017. New York: United Nations. Available from: https://esa.un.org/unpd/wpp/Download/Standard/Population/. Cited 15.05.2018.
