# Supplementary material for: Cost-effectiveness analyses and cost analyses in castration-resistant prostate cancer: A systematic review
Source: PLoS One. 2018 Dec 5;13(12):e0208063. doi: 10.1371/journal.pone.0208063 (PMC6281264; doi:10.1371/journal.pone.0208063)
Supplement: S2 Table — ✓: Criterion fulfilled; CHEERS: consolidated health economic evaluation reporting standards, n.a.: not applicable. (PDF) [file pone.0208063.s002.pdf]

S2 Table. Quality assessment of included cost-effectiveness analyses (based on the CHEERS-checklist [40])

Reference	Andronis [57]	Bloomfield [58]	James [59]	Reed [60]	Studies that fulfilled the respective criterion (%)
Title and abstract					
Title	✓	✓	✓	✓	100%
Abstract	✓	✓	✓	✓	100%
Introduction					
Background and objectives	✓	✓	✓	✓	100%
Methods					
Target population and subgroups	✓	✓	✓	✓	100%
Setting and location		✓			25%
Study perspective	✓	✓	✓	✓	100%
Comparators	✓	✓	✓	✓	100%
Time horizon	✓	✓	✓	✓	100%
Discount rate	✓	✓	✓		75%
Choice of health outcomes	✓	✓	✓	✓	100%
Measurement of effectiveness	✓	✓	✓	✓	100%
Measurement and valuation of preference based outcomes	✓		✓	✓	75%
Estimating resources and costs	✓	✓	✓	✓	100%
Currency, price date, and conversion		✓	✓	✓	75%
Choice of model	n.a.	n.a.	n.a.	n.a.	–
Assumptions	n.a.	n.a.	n.a.	n.a.	–
Analytical methods	✓		✓		50%
Results					
Study parameters	✓	✓	✓		75%
Incremental costs and outcomes	✓	✓	✓	✓	100%
Characterizing uncertainty	✓	✓	✓	✓	100%
Characterizing heterogeneity	n.a.	n.a.	n.a.	n.a.	–
Discussion					
Study findings, limitations, generalizability, and current knowledge	✓	✓	✓	✓	100%
Other					
Source of funding			✓	✓	50%
Conflicts of interest	✓		✓	✓	75%
**Criteria each study fulfilled (%)**	86%	81%	95%	81%	

✓: Criterion fulfilled; CHEERS: consolidated health economic evaluation reporting standards, n.a.: not applicable.
