# Supplementary material for: Cost-effectiveness analyses and cost analyses in castration-resistant prostate cancer: A systematic review
Source: PLoS One. 2018 Dec 5;13(12):e0208063. doi: 10.1371/journal.pone.0208063 (PMC6281264; doi:10.1371/journal.pone.0208063)
Supplement: S3 Table — ✓: Criterion fulfilled, CHEERS: consolidated health economic evaluation reporting standards, n.a.: not applicable. (PDF) [file pone.0208063.s003.pdf]

S3 Table. Quality assessment of included model-based economic evaluations (based on the CHEERS-checklist [40])

Reference	Carter [61]	Collins [56]	Gong [62]	Holko [63]	Konski [64]	Massoudi [65]	Peters [55]	Pilon [66]	Pollard [67]	Sne-decor [68]	Stop-eck [69]	Wilson [70]	Xie [71]	Zhong [72]	Zubek [73]	Studies that fulfilled the respective criterion (%)
Title and abstract																
Title	✓	✓	✓	✓					✓	✓	✓		✓		✓	60%
Abstract	✓		✓	✓	✓	✓	✓	✓	✓	✓	✓	✓	✓	✓	✓	93%
Introduction																
Background and objectives	✓	✓	✓	✓	✓	✓	✓	✓	✓	✓	✓	✓	✓	✓	✓	100%
Methods																
Target population and subgroups	✓	✓	✓	✓	✓	✓		✓	✓	✓	✓		✓	✓		80%
Setting and location	✓	✓	✓										✓			27%
Study perspective	✓	✓	✓	✓	✓	✓	✓	✓		✓	✓	✓	✓		✓	87%
Comparators	✓	✓	✓	✓	✓	✓	✓	✓		✓	✓	✓	✓	✓	✓	93%
Time horizon	✓	✓	✓	✓	✓	✓	✓			✓	✓	✓	✓	✓	✓	87%
Discount rate		✓	✓	✓			✓		n.a.	✓	✓	n.a.	✓	n.a.	✓	67%
Choice of health outcomes	✓	✓	✓	✓	✓	✓	✓	✓	✓	✓	✓	✓	✓	✓	✓	100%
Measurement of effectiveness	✓	✓	✓	✓	✓		✓							✓		47%
Measurement and valuation of preference based outcomes	✓	✓	✓	✓	✓	n.a.	✓	n.a.	n.a.	✓	✓	n.a.	n.a.	n.a.	n.a.	100%
Estimating resources and costs	✓	✓	✓	✓	✓	✓	✓	✓	✓	✓	✓	✓	✓	✓	✓	100%

Currency, price date, and conversion	✓	✓	✓	✓		✓	✓			✓	✓	✓	✓	✓	✓	80%
Choice of model			✓				✓			✓	✓		✓		✓	40%
Assumptions			✓		✓		✓			✓	✓	✓	✓	✓	✓	60%
Analytical methods		✓	✓	✓			✓			✓	✓	✓	✓	✓	✓	67%
Results																
Study parameters	✓	✓	✓	✓	✓	✓	✓	✓	✓	✓	✓	✓	✓	✓	✓	100%
Incremental costs and outcomes	✓	✓		✓	✓	✓	✓	✓		✓	✓	✓	✓	✓	✓	87%
Characterizing uncertainty	✓	✓	✓	✓	✓	✓	✓	✓		✓	✓	✓	✓	✓	✓	93%
Characterizing heterogeneity	n.a.	n.a.	n.a.	n.a.	n.a.	n.a.	n.a.	n.a.	n.a.	n.a.	n.a.	n.a.	n.a.	n.a.	n.a.	–
Discussion																
Study findings, limitations, generalizability, and current knowledge	✓	✓	✓	✓	✓	✓	✓	✓	✓	✓	✓	✓	✓	✓	✓	100%
Other																
Source of funding	✓	✓				✓	✓	✓		✓	✓	✓	✓	✓	✓	73%
Conflicts of interest	✓		✓	✓		✓	✓	✓	✓	✓	✓			✓	✓	73%
**Criteria each study fulfilled (%)**	83%	83%	91%	83%	65%	68%	87%	59%	43%	91%	91%	71%	91%	81%	86%	

✓: Criterion fulfilled, CHEERS: consolidated health economic evaluation reporting standards, n.a.: not applicable.
