# Supplementary material for: Cost-effectiveness analyses and cost analyses in castration-resistant prostate cancer: A systematic review
Source: PLoS One. 2018 Dec 5;13(12):e0208063. doi: 10.1371/journal.pone.0208063 (PMC6281264; doi:10.1371/journal.pone.0208063)
Supplement: S4 Table — ✓: Criterion fulfilled, n.a.: not applicable. (PDF) [file pone.0208063.s004.pdf]

S4 Table. Quality assessment of included cost-of-illness analyses (based on Stuhldreher et al. [44])

Reference	Alemayehu [74]	Armstrong [75]	Bourke [76]	Bryant-Lukosius [77]	Bui [78]	Dragomir [79]	Engel-Nitz [80]	Krahn [81]	Kunisawa [82]	Malmberg [83]	Mehra [84]	Organ [85]	Sanyal [86]	Satoh [55]	Sherman [87]	Studies that fulfilled the respective criterion (%)
Scope																
Study objective	✓	✓	✓	✓	✓	✓	✓	✓	✓	✓	✓	✓	✓	✓	✓	100%
Inclusion and exclusion criteria	✓	✓		✓	✓		✓	✓	✓			✓			✓	60%
Disease and diagnostic criteria	✓	✓			✓		✓	✓			✓			✓		47%
General economic																
Cost-description	✓	✓	✓	✓	✓	✓	✓	✓	✓	✓	✓	✓	✓	✓	✓	100%
Non-diseased comparison group or disease-specific costs	✓					✓				✓			✓			27%
Calculation of costs																
Currency	✓	✓	✓	✓	✓	✓	✓	✓	✓	✓	✓	✓	✓	✓	✓	100%
Reference year	✓	✓	✓	✓	✓	✓	✓	✓	✓	✓		✓	✓			80%
Perspective	✓	✓	✓	✓		✓					✓		✓			47%
Costs incorporated from more than one category	✓	✓	✓	✓	✓	✓	✓	✓	✓	✓	✓	✓	✓	✓	✓	100%
Data source	✓	✓	✓	✓	✓	✓	✓	✓	✓	✓	✓	✓	✓	✓	✓	100%
Valuation of costs	n.a.	n.a.	n.a.	✓	n.a.	n.a.	n.a.	✓	n.a.	n.a.	n.a.	n.a.	n.a.	n.a.	✓	100%
Discounting			n.a.	n.a.						✓			✓		n.a.	17%
Study design and analysis																

Missing data and imputation method	n.a.	n.a.	n.a.	n.a.	n.a.	n.a.	n.a.	✓		n.a.	n.a.	n.a.	n.a.	n.a.	n.a.	50%
Statistics appropriate	✓			✓	✓	✓	✓	✓		✓	✓		✓		✓	67%
Sensitivity analyses						✓			✓				✓			20%
Presentation of results																
Sample size (subgroup)	✓	✓	n.a.	✓	✓	✓	✓	✓	✓	✓	✓	✓	n.a.	✓	✓	100%
Demographics	✓				✓	n.a.			✓		✓	✓	n.a.	✓	✓	54%
Arithmetic mean costs	✓	✓	✓	✓	✓	✓	✓	✓	✓	✓	✓	✓	✓	✓	✓	100%
Standard deviations (errors)	✓		✓	✓	✓	✓			✓				✓	✓		53%
Discussion																
Results discussed with respect to other studies	✓	✓		✓		✓	✓	✓	✓	✓	✓	✓	✓	✓	✓	87%
Limitations discussed	✓	✓	✓	✓	✓	✓	✓	✓	✓	✓	✓	✓	✓	✓	✓	100%
Conclusions appropriate regarding uncertainty in results	✓	✓	✓	✓		✓	✓	✓	✓	✓	✓	✓	✓	✓	✓	93%
**Criteria each study fulfilled (%)**	90%	70%	61%	80%	70%	84%	70%	73%	71%	70%	70%	65%	89%	65%	70%	

✓: Criterion fulfilled, n.a.: not applicable.
