# Supplementary material for: Cost-effectiveness analyses and cost analyses in castration-resistant prostate cancer: A systematic review
Source: PLoS One. 2018 Dec 5;13(12):e0208063. doi: 10.1371/journal.pone.0208063 (PMC6281264; doi:10.1371/journal.pone.0208063)
Supplement: S5 Table — ✓: Criterion fulfilled, (✓): criterion partially fulfilled, ECOBIAS: Bias in Economic Evaluation, n.a.: not applicable. (PDF) [file pone.0208063.s005.pdf]

S5 Table. Risk of bias assessment of included cost-effectiveness analyses (based on the ECOBIAS checklist [45])

**Reference**	**Andronis [57]**	**Bloomfield [58]**	**James [59]**	**Reed [60]**	**Studies that fulfilled the respective criterion (%)**
Narrow perspective bias		✓	✓	✓	75%
Inefficient comparator bias	✓	✓	✓	✓	100%
Cost measurement omission bias	✓	✓	✓		75%
Intermittent data collection bias	✓	✓	✓	✓	100%
Invalid valuation bias	✓	✓	✓	(✓)	75%
Ordinal ICER bias	✓	✓	✓	✓	100%
Double-counting bias	n.a.	n.a.	n.a.	n.a.	–
Inappropriate discounting bias	✓	✓	✓	(✓)	75%
Limited sensitivity analysis bias		(✓)	✓	(✓)	25%
Sponsor bias			✓	(✓)	25%
Reporting and dissemination bias	(✓)	(✓)	✓		25%
**Criteria each study fulfilled (%)**	60%	70%	100%	40%	

✓: Criterion fulfilled, (✓): criterion partially fulfilled, ECOBIAS: Bias in Economic Evaluation, n.a.: not applicable.
