# Supplementary material for: Cost-effectiveness analyses and cost analyses in castration-resistant prostate cancer: A systematic review
Source: PLoS One. 2018 Dec 5;13(12):e0208063. doi: 10.1371/journal.pone.0208063 (PMC6281264; doi:10.1371/journal.pone.0208063)
Supplement: S6 Table — ✓: Criterion fulfilled, (✓): criterion partially fulfilled, ECOBIAS: Bias in Economic Evaluation, n.a.: not applicable. * These biases are overlapping regarding their content, ** these biases are overlapping regarding their content. (PDF) [file pone.0208063.s006.pdf]

S6 Table. Risk of bias assessment of included model-based economic evaluations (based on the ECOBIAS checklist [45])

Reference	Carter [61]	Collins [56]	Gong [62]	Holko [63]	Konski [64]	Massoudi [65]	Peters [55]	Pilon [66]	Pollard [67]	Sne-decor [68]	Stop-eck [69]	Wilson [70]	Xie [71]	Zhong [72]	Zubek [73]	Studies that fulfilled the respective criterion (%)
Narrow perspective bias			✓				✓		✓			✓				27%
Inefficient comparator bias*	✓	✓	✓	✓	✓	✓	✓	✓		✓	✓	✓	✓	✓	✓	93%
Cost measurement omission bias		✓	✓	✓		✓	✓			✓	✓	✓	✓	✓		67%
Intermittent data collection bias	n.a.	n.a.	n.a.	n.a.	n.a.	n.a.	n.a.	n.a.	n.a.	n.a.	n.a.	n.a.	n.a.	n.a.	n.a.	–
Invalid valuation bias	✓	✓	✓	✓		✓				✓	✓	✓	✓	✓	✓	73%
Ordinal ICER bias	✓	✓	✓	✓	✓	✓	✓	n.a.	✓	✓	✓	✓	✓	✓	✓	100%
Double-counting bias	n.a.	n.a.	n.a.	n.a.	n.a.	n.a.	n.a.	n.a.	n.a.	n.a.	n.a.	n.a.	n.a.	n.a.	n.a.	–
Inappropriate discounting bias		✓	✓	✓		✓	✓		n.a.	✓	✓	n.a.	✓	n.a.	✓	75%
Limited sensitivity analysis bias**	(✓)	(✓)	(✓)	(✓)	(✓)	(✓)	(✓)			(✓)	(✓)	(✓)	(✓)	(✓)	(✓)	0%
Sponsor bias		(✓)		(✓)		(✓)	(✓)	(✓)		(✓)	(✓)	(✓)	(✓)	(✓)	(✓)	0%
Reporting and dissemination bias	n.a.	n.a.	n.a.	n.a.	n.a.	n.a.	n.a.	n.a.	n.a.	n.a.	n.a.	n.a.	n.a.	n.a.	n.a.	–
Bias related to structure																
Structural assumptions bias	✓	✓	✓	✓	✓	✓	✓		✓	✓	✓	✓	✓	✓	✓	93%

No treatment comparator bias*	✓	✓	✓	✓	✓	✓	✓	✓		✓	✓	✓	✓	✓	✓	93%
Wrong model bias		✓	✓	✓	✓		✓			✓	✓		✓		✓	60%
Limited time horizon bias		✓	✓	✓	✓		✓	✓		✓	✓		✓	✓	✓	73%
Bias related to data																
Bias related to data identification	✓	✓		✓	✓		✓									33%
Bias related to baseline data	✓	✓	✓	✓	✓	✓	✓	✓	(✓)	✓	✓	✓	✓	✓	✓	93%
Bias related to treatment effects	✓	✓	✓	✓		✓	✓	n.a.	n.a.	n.a.	n.a.	n.a.	n.a.	n.a.	n.a.	86%
Bias related to quality-of-life weights (utilities)	✓	✓	✓	✓	✓	n.a.	✓	n.a.	n.a.	✓	✓	✓	n.a.	✓	✓	100%
Non-transparent data incorporation bias	✓	✓	✓		✓		✓			✓	✓	✓	✓	✓	✓	73%
Limited scope bias**	(✓)	(✓)	(✓)	(✓)	(✓)	(✓)	(✓)			(✓)	(✓)	(✓)	(✓)	(✓)	(✓)	0%
Bias related to consistency																
Bias related to internal consistency																0%
**Criteria each study fulfilled (%)**	53%	74%	74%	68%	53%	50%	74%	25%	19%	67%	67%	59%	65%	59%	61%	

✓: Criterion fulfilled, (✓): criterion partially fulfilled, ECOBIAS: Bias in Economic Evaluation, n.a.: not applicable.

* These biases are overlapping regarding their content, ** these biases are overlapping regarding their content.
