# Supplementary material for: Cost-effectiveness analyses and cost analyses in castration-resistant prostate cancer: A systematic review
Source: PLoS One. 2018 Dec 5;13(12):e0208063. doi: 10.1371/journal.pone.0208063 (PMC6281264; doi:10.1371/journal.pone.0208063)
Supplement: S2 File — (PDF) [file pone.0208063.s008.pdf]

References of the excluded non-relevant articles

1. Exisulind: Aptosyn, FGN 1, Prevatac, sulindac sulfone. Drugs R D. 2004;5: 220-226.
2. Satraplatin: BMS 182751, BMY 45594, JM 216. Drugs R D. 2007;8: 125-132.
3. Abstracts of the 2nd Prostate Cancer World Congress. BJU Int. 2015;116 Suppl 1. doi: 10.1111/bju.13196
4. Akl EA, Kahale LA, Ballout RA, Barba M, Yosuico VED, van Doormaal FF, et al. Parenteral anticoagulation in ambulatory patients with cancer. Cochrane Database Syst Rev. 2014. doi: 10.1002/14651858.CD006652.pub4
5. Albers P, Heicappell R, Schwaibold H, Wolff J, German Association of Urological Oncology SotGCS. Erythropoietin in urologic oncology. Eur Urol. 2001;39: 1-8. doi: 52404
6. Alemayehu B, Parry D, Engel-Nitz NM, Kulakodlu M, Nathan F. Costs at the end of life for patients with castration-resistant prostate cancer (CRPC). Value in Health. 2011;14: A161-A162.
7. Alice D, Rocha J, Aprikian A, Vanhuyse M, Cury F, Kassouf W. Use of abiraterone acetate in the management of castration-resistant prostate cancer: a real-life cost effectiveness study. J Urol. 2016;195: E624-E624.
8. Armstrong AJ, Bui CN, Fitch K, Sawhney T, Brown B, Flanders SC, et al. Docetaxel chemotherapy in metastatic castration-resistant prostate cancer (mCRPC): Cost of care for Medicare and commercially insured men. J Clin Oncol. 2016;34: 1133-1139. doi: 10.1080/03007995.2017.1308919
9. Aronson N, Seidenfeld J. Relative effectiveness and cost-effectiveness of methods of androgen suppression in the treatment of advanced prostate cancer (Structured abstract). Database of Abstracts of Reviews of Effects. 1999: 246.

10. Arrabal-Martín M, Anglada-Curado F, Cózar-Olmo JM, Soler-Martínez J, Moreno-Jiménez J, Castiñeiras-Fernández J, et al. Pre-chemotherapy abiraterone acetate. A proposal of a treatment algorithm in castration resistant prostate cancer. Actas Urol Esp. 2014;38: 327-333. doi: 10.1016/j.acuro.2013.10.008

11. Attard G, Parker C, Eeles RA, Schröder F, Tomlins SA, Tannock I, et al. Prostate cancer. Lancet. 2016;387 North American Edition: 70-82. doi: 10.1016/S0140-6736(14)61947-4

12. Basch E, Autio K, Ryan CJ, Mulders P, Shore N, Kheoh T, et al. Abiraterone acetate plus prednisone versus prednisone alone in chemotherapy-naive men with metastatic castration-resistant prostate cancer: patient-reported outcome results of a randomised phase 3 trial. Lancet Oncol. 2013;14: 1193-1199. doi: 10.1016/S1470-2045(13)70424-8

13. Berger J, Dunn JD, Johnson MM, Karst KR, Shear WC. How drug life-cycle management patent strategies may impact formulary management. Am J Manag Care. 2016: S487-S495.

14. Berlin JD, Propert KJ, Trump D, Wilding G, Hudes G, Glick J, et al. 5-Fluorouracil and leucovorin therapy in patients with hormone refractory prostate cancer: an Eastern Cooperative Oncology Group phase II study (E1889). Am J Clin Oncol. 1998;21: 171-176.

15. Bitting RL, Armstrong AJ. Potential predictive biomarkers for individualizing treatment for men with castration-resistant prostate cancer. Cancer J. 2013;19: 25-33. doi: 10.1097/PPO.0b013e31827e0b9c

16. Breton X, Lechevallier E, Coulange C. [Current place of chemotherapy in the treatment of hormone-refractory metastatic prostate cancer]. Prog Urol. 2005;15: 398-404.

17. Brundage MD, Crook JM, Lukka H. Use of strontium-89 in patients with endocrine-refractory carcinoma of the prostate metastatic to bone (Structured abstract). Database of Abstracts of Reviews of Effects. 1997.

18. Buchan NC, Goldenberg SL. Intermittent androgen suppression for prostate cancer. Nat Rev Urol. 2010;7: 552-560. doi: 10.1038/nrurol.2010.141
19. Buroni FE, Persico MG, Pasi F, Lodola L, Nano R, Aprile C. Radium-223: insight and perspectives in bone-metastatic castration-resistant prostate cancer. Anticancer Res. 2016;36: 5719-5730. doi: 10.21873/anticanres.11155
20. Caffo O, Maines F, Rizzo M, Kinspergher S, Veccia A. Metastatic castration-resistant prostate cancer in very elderly patients: challenges and solutions. Clin Interv Aging. 2017;12: 19-28. doi: 10.2147/cia.s98143
21. Canadian Agency for Drugs and Technologies in Health. Radium-223 for Patients with castration resistant prostate cancer with bone metastases: a review of clinical effectiveness, cost-effectiveness and guidelines. Ottawa: Canadian Agency for Drugs and Technologies in Health; 2016.
22. Cervera JM, Garcia-Carbonero I, Girones R, Beltran M, Calderero V, Gonzalvez ML, et al. Clinical experience with oral vinorelbine (NVB) plus prednisone as first- or second-line chemotherapy of metastatic hormone-refractory prostate cancer (HRPC). J Clin Oncol. 2009;27: e16151. doi: 10.1200/jco.2009.27.15s.e16151
23. Chambers SK, Occhipinti S, Foley E, Clutton S, Legg M, Berry M, et al. Mindfulness-based cognitive therapy in advanced prostate cancer: a randomized controlled trial. J Clin Oncol. 2017;35: 291-297. doi: 10.1200/jco.2016.68.8788
24. Cherny N, Sullivan R, Torode J, Saar M, Eniu A. ESMO European Consortium Study on the availability, out-of-pocket costs and accessibility of antineoplastic medicines in Europe. Ann Oncol. 2016;27: 1423-1443. doi: 10.1093/annonc/mdw213
25. Chopra A, Gluck S, Montero AJ, Avancha K, Lopes G. Cost-effectiveness evaluation of abiraterone in the treatment of patients with castration-resistant prostate cancer who previously received docetaxel. J Clin Oncol. 2012;30. doi: 10.1200/jco.2012.30.15_suppl.e15107

26. Choudhury PS, Gupta M. Personalized and precision medicine in cancer : a theragnostic approach. Curr Radiopharm. 2017;10: 166-170. doi: 10.2174/1874471010666170728094008

27. Chuu C-P, Kokontis JM, Hiipakka RA, Fukuchi J, Lin H-P, Lin C-Y, et al. Androgens as therapy for androgen receptor-positive castration-resistant prostate cancer. J Biomed Sci. 2011;18: 63. doi: 10.1186/1423-0127-18-63

28. Collins R, Fenwick E, Trowman R, Perard R, Norman G, Light K, et al. A systematic review and economic model of the clinical effectiveness and cost-effectiveness of docetaxel in combination with prednisone or prednisolone for the treatment of hormone-refractory metastatic prostate cancer. Health Technol Assess. 2007;11: 1-198. doi: 10.3310/hta11020

29. Colloca G, Colloca P. Health-related quality of life assessment in prospective trials of systemic cytotoxic chemotherapy for metastatic castration-resistant prostate cancer: which instrument we need? Med Oncol. 2011;28: 519-527. doi: 10.1007/s12032-010-9495-2

30. Crawford ED, Shore ND, Petrylak DP, Higano CS, Ryan CJ. Abiraterone acetate and prednisone in chemotherapy-naïve prostate cancer patients: rationale, evidence and clinical utility. Ther Adv Med Oncol. 2017;9: 319-333. doi: 10.1177/1758834017698644

31. da Silva FC. Intermittent hormonal therapy for prostate cancer. Curr Opin Urol. 2011;21: 248-251. doi: 10.1097/MOU.0b013e328344f3e3

32. De CP, Atallah ÁN, Arruda HO, Soares BG, El DRP, Wilt TJ. Intermittent versus continuous androgen suppression for prostatic cancer. Cochrane Database Syst Rev. 2007. doi: 10.1002/14651858.CD005009.pub2

33. de Meijer C, Baka A, Leliveld-Kors A, Noordzij W, Gaultney J. Cost-effectiveness of radium-223 compared to best standard of care, abiraterone acetate and enzalutamide in the treatment of castration resistant prostate cancer in the Netherlands. Eur J Cancer. 2015;51: S179-S180.

34. Dearden L, Girod I, Majer I, van de Wetering G. A cost comparison of treatment with abiraterone acetate plus prednisone in the pre chemotherapy setting followed by enzalutamide in the post-chemotherapy setting versus the opposite treatment sequence in metastatic castration resistant prostate cancer patients with non-visceral metastases. Value Health. 2015;18: A446-A446. doi: 10.1016/j.jval.2015.09.1109
35. Dellavedova T, Boetto M, Olmedo J, Sarria JP, Nóbile R, Ponzano R, et al. Enzalutamide as monotherapy for advanced prostate cancer: why not? Arch Esp Urol. 2016;69: 393-397.
36. Dellis A, Papatsoris A. Cost-effectiveness of denosumab as a bone protective agent for patients with castration resistant prostate cancer. Expert Rev Pharmacoecon Outcomes Res. 2016;16: 5-10. doi: 10.1586/14737167.2016.1123624
37. Dellis A, Papatsoris AG. Denosumab as a promising novel bone-targeted agent in castration resistant prostate cancer. Expert Opin Biol Ther. 2014;14: 7-10. doi: 10.1517/14712598.2013.840582
38. Demir U, Koehler A, Schneider R, Schweiger S, Klocker H. Metformin anti-tumor effect via disruption of the MID1 translational regulator complex and AR downregulation in prostate cancer cells. BMC Cancer. 2014;14: 52. doi: 10.1186/1471-2407-14-52
39. Dhillon S, Lyseng-Williamson KA. Zoledronic acid - A review of its use in the management of bone metastases of malignancy. Drugs. 2008;68: 507-534. doi: 10.2165/00003495-200868040-00010
40. di Lorenzo G, Ferro M, Buonerba C. Sipuleucel-T (Provenge®) for castration-resistant prostate cancer. BJU Int. 2012;110: E99-E104. doi: 10.1111/j.1464-410X.2011.10790.x
41. Di NM, Porreca E, Candeloro M, De TM, Russi I, Rutjes AW. Primary prophylaxis for venous thromboembolism in ambulatory cancer patients receiving chemotherapy. Cochrane Database Syst Rev. 2016. doi: 10.1002/14651858.CD008500.pub4

42. Dragomir A, Dinea D, Vanhuyse M, Cury F, Aprikian A. Metastatic Castration-Resistant Prostate Cancer: Treatment Pathway and Associated Cost in Canada. Pharmacoepidemiol Drug Saf. 2013;22: 419-419. doi: 10.1186/1472-6963-14-252

43. Dragomir A, Vanhuyse M, Aprikian A. Cost comparison of different forms of androgen ablative therapies in metastatic castration-resistant prostate cancer in Canada. Value Health. 2014;17: A78-A78.

44. Dragomir A, Vanhuyse M, Cury FL, Aprikian AG. Canadian cost comparison of different forms of androgen ablative therapies prior and during the castration-resistant prostate cancer. J Clin Oncol. 2014;32. doi: 10.1200/jco.2014.32.15_suppl.e16029

45. Duran A, Watt M, Flanders S, Schultz NM. Number needed to treat and associated incremental costs of treatment with enzalutamide versus abiraterone acetate plus prednisone in chemotherapy-naïve patients with metastatic castration-resistant prostate cancer in Spain and the United Kingdom. Value Health. 2017;20: A107-A107.

46. Dyer M, Rinaldi F, George E, Adler AI, Dyer M, Rinaldi F, et al. NICE guidance on abiraterone for castration-resistant metastatic prostate cancer previously treated with a docetaxel-containing regimen. Lancet Oncol. 2012;13: 762-763. doi: 10.1016/S1470-2045(12)70289-9

47. Efstathiou E, Gyftaki R, Kousoulakou H, Paparouni K, Ikonomou V. Abiraterone Acetate Versus Cabazitaxel in the Treatment of Metastatic Castration-Resistant Prostate Cancer: An Economic Evaluation in the Greek Health Care Setting. Value Health. 2012;15: A424. doi: 10.1016/j.jval.2012.08.2124

48. Ellis LA, Lafeuille M-H, Gozalo L, Pilon D, Lefebvre P, McKenzie S. Treatment sequences and pharmacy costs of 2 new therapies for metastatic castration-resistant prostate cancer. Am Health Drug Benefits. 2015;8: 185-194.

49. Engels FK, Verweij J. Docetaxel administration schedule: From fever to tears? A review of randomised studies. Eur J Cancer. 2005;41: 1117-1126. doi: 10.1016/j.ejca.2005.02.016

50. Esper P, Hampton JN, Finn J, Smith DC, Regiani S, Pienta KJ. A new concept in cancer care: the supportive care program. Am J Hosp Palliat Care. 1999;16: 713-722.

51. Facchini G, Perri F, Misso G, D Aniello C, Scarpati GDV, Rossetti S, et al. Optimal management of prostate cancer based on its natural clinical history. Curr Cancer Drug Targets. 2017. doi: 10.2174/1568009617666170209093101

52. Fizazi K, Albiges L, Massard C, Escudier B, Loriot Y. Novel and bone-targeted agents for CRPC. Ann Oncol. 2012;23 Suppl 10: x264-267. doi: 10.1093/annonc/mds353

53. Foxon G, Fox G, Craddy P. Do ayers equally value the cost per month of overall survival for metastatic castration resistant prostate cancer within the Eu5? Value Health. 2015;18: A444. doi: 10.1016/j.jval.2015.09.1100

54. Fujikawa K, Matsui Y, Fukuzawa S, Takeuchi H. Prostate-specific antigen levels and clinical response to flutamide as the second hormone therapy for hormone-refractory prostate carcinoma. Eur Urol. 2000;37: 218-222.

55. Gamboa O, Bonilla C, Varela R. A cost comparison of treatment with abiraterone acetate plus prednisone in the pre-chemotherapy setting followed by enzalutamide in the post-chemotherapy setting versus the opposite treatment sequence in metastatic castration resistant prostate cancer patients with non-visceral metastases in Colombia. Value Health. 2017;20: A100.

56. García de Paredes Esteban JC, Alegre Del Rey EJ, Asensi Díez R. Docetaxel in hormone-sensitive advanced prostate cancer; GENESIS-SEFH evaluation report. Farm Hosp. 2017;41: 550-558. doi: 10.7399/fh.2017.41.4.10742

57. Gaultney J, Baka A, Leliveld-Kors A, Noordzij W, Wyndaele D, De Meyer C. Results of a Dutch cost-effectiveness model of eadium-223 in comparison to cabazitaxel, abiraterone, and enzalutamide in patients with metastatic castration resistant prostate cancer previously treated with docetaxel. Value Health. 2015;18: A459. doi: 10.1016/j.jval.2015.09.1184

58. Ghatnekar O, Nørgaard K, Skaltsa K. Results and implications of using a new eq-5d value set for cost-utility analyses in sweden. An application using enzalutamide (xtandi®) verse best supportive care for treatment of metastatic castration resistant prostate cancer (MCRPC). Value Health. 2014;17: A648. doi: 10.1016/j.jval.2014.08.2350

59. Gibbons RP. Localized prostate carcinoma. Surgical management. Cancer. 1993;72: 2865-2812.

60. Goethuys H, Baert L, Van Poppel H, Lieskovsky G, Brady LW, Petrovich Z. Treatment of metastatic carcinoma of the prostate. Am J Clin Oncol. 1997;20: 40-45.

61. Goetz D. New options for the management of castration-resistant prostate cancer: a case perspective. J Natl Compr Canc Netw. 2011;9: S13-S24. doi: 10.6004/jnccn.2011.0128

62. Goldberg T, Berrios-Colon E. Abiraterone (Zytiga), a novel agent for the management of castration-resistant prostate cancer. P T. 2013;38: 23-26.

63. Goluboff ET, Hirano D, Thrasher JB, Stark G, Miller GJ, Glodé LM. New approaches to the treatment of advanced prostate cancer. Rev Urol. 2001;3 Suppl 2: S69-S78.

64. Graff JN, Beer TM. Reducing Skeletal-Related Events in Metastatic Castration-Resistant Prostate Cancer. Oncology (Williston Park). 2015;29: 416-423.

65. Group IW, Sweeney C, Nakabayashi M, Regan M, Xie W, Hayes J, et al. The development of intermediate clinical endpoints in cancer of the prostate (ICECaP). J Natl Cancer Inst. 2015;107: djv261. doi: 10.1093/jnci/djv261

66. Gulley J, Madan R, Schlom J. Current status of recombinant pox-viral vaccines. Cancer Res. 2012;72. doi: 10.1158/1538-7445.AM2012-PL03-02

67. Haddad H, Garcia JA. Novel agents for the management of castration-resistant prostate cancer. Curr Opin Urol. 2012;22: 175-182. doi: 10.1097/MOU.0b013e3283523ba0

68. Hammerstrom AE, Cauley DH, Atkinson BJ, Sharma P. Cancer immunotherapy: sipuleucel-T and beyond. Pharmacotherapy. 2011;31: 813-828. doi: 10.1592/phco.31.8.813

69. Haseen F, Cantwell MM, O'Sullivan JM, Murray LJ. Is there a benefit from lycopene supplementation in men with prostate cancer? A systematic review. Prostate Cancer Prostatic Dis. 2009;12: 325-332. doi: 10.1038/pcan.2009.38

70. He J, Li T, Saadi R. Abiraterone acetate versus enzalutamide for metastatic castration-resistant prostate cancer post chemotherapy: cost effectiveness analysis. Value Health. 2013;16: A411. doi: 10.1016/j.jval.2013.08.506

71. Heitzer E, Ulz P, Belic J, Gutschi S, Quehenberger F, Fischereder K, et al. Tumor-associated copy number changes in the circulation of patients with prostate cancer identified through whole-genome sequencing. Genome Med. 2013;5: 30. doi: 10.1186/gm434

72. Henricks P, Cislo P, Zhan L, Beaudet A, Grabbi E, Lloyd A, et al. Cost-effectiveness of radium-223 dichloride (radium-223) in ALSYMPCA: a cost-effectiveness analysis fo radium-223 + best standard of care (BSOC) compared with placebo plus BSOC in treatment of castration-resistant prostate cancer (CRPC) and symptomatic bone metastases in Canada. Value Health. 2015;18: A2024.

73. Higano CS. Understanding treatments for bone loss and bone metastases in patients with prostate cancer: a practical review and guide for the clinician. Urol Clin North Am. 2004;31: 331-352. doi: 10.1016/j.ucl.2004.01.001

74. Higgins GS, Stewart GD, McNeill SA, McLaren DB. A long and winding road: the role of chemotherapy for hormone-refractory prostate cancer. Int J Clin Pract. 2007;61: 1964-1965. doi: 10.1111/j.1742-1241.2007.01550.x

75. Homedes N, Ugalde A. Health and ethical consequences of outsourcing pivotal clinical trials to Latin America: a cross-sectional, descriptive study. PloS one. 2016;11: e0157756. doi: 10.1371/journal.pone.0157756

76. Hoy SM. Abiraterone acetate: a review of its use in patients with metastatic castration-resistant prostate cancer. Drugs. 2013;73: 2077-2091. doi: 10.1007/s40265-013-0150-z

77. Iranikhah M, Wilborn TW, Wensel TM, Ferrell JB. Denosumab for the prevention of skeletal-related events in patients with bone metastasis from solid tumor. Pharmacotherapy. 2012;32: 274-284. doi: 10.1002/j.1875-9114.2011.01092.x

78. Ivanescu C, Skaltsa K, Holmstrom S. Mapping the disease-specific fact-P to the preference-based EQ-5D in castration-resistant prostate cancer. Value Health. 2012;15: A529-A530. doi: 10.1016/j.jval.2012.08.1838

79. Jakob JJ, Schmidt S, Kunath F, Meerpohl JJ, Blümle A, Schmucker C, et al. Degarelix for treating advanced hormone-sensitive prostate cancer. Cochrane Database Syst Rev 2017. doi: 10.1002/14651858.CD012548

80. James ND, Bloomfield D, Luscombe C. The changing pattern of management for hormone-refractory, metastatic prostate cancer. Prostate Cancer Prostatic Dis. 2006;9: 221-229. doi: 10.1038/sj.pcan.4500880

81. Jessome R. Ra-223 alpha-therapy in pPatients with bone metastases from castration-resistant prostate cancer. J Med Imaging Radiat Oncol. 2015;46: 156-161. doi: 10.1016/j.jmir.2015.01.003

82. Joulain F, Zavisic S, Mehta J. Cabazitaxel In Second Line (2l) Treatment Of Metastatic Castration Resistant Prostate Cancer : An Economic Evaluation In Sweden. Value Health. 2013;16: A138. doi: 10.1016/j.jval.2013.03.676

83. Kalkner KM, Westlin JE, Strang P. 89Strontium in the management of painful sceletal metastases. Anticancer Res. 2000;20: 1109-1114.

84. Karan D, Holzbeierlein JM, Van Veldhuizen P, Thrasher JB. Cancer immunotherapy: a paradigm shift for prostate cancer treatment. Nat Rev Urol. 2012;9: 376-385. doi: 10.1038/nrurol.2012.106

85. Kostyuk A, Akanov A. Cost Effectiveness of New Therapeutic Options in Castration-Resistant Metastatic Prostate Cancer. Value Health. 2016;19: A151. doi: 10.1016/j.jval.2016.03.1596

86. Kubler H, Scheel B, Gnad-Vogt U, Miller K, Schultze-Seemann W, Dorp F, et al. Self-adjuvanted mRNA vaccination in advanced prostate cancer patients: a first-in-man phase I/IIa study. J Immunother Cancer. 2015;3: 26. doi: 10.1186/s40425-015-0068-y

87. Laajala TD, Jumppanen M, Huhtaniemi R, Fey V, Kaur A, Knuuttila M, et al. Optimized design and analysis of preclinical intervention studies in vivo. Sci Rep. 2016;6: 30723. doi: 10.1038/srep30723

88. Ladoire S, Eymard JC, Zanetta S, Mignot G, Martin E, Kermarrec I, et al. Metronomic oral cyclophosphamide prednisolone chemotherapy is an effective treatment for metastatic hormone-refractory prostate cancer after docetaxel failure. Anticancer Res. 2010;30: 4317-4323.

89. Le TK, Zhang Y, Zyczynski TM. Cost Of Metastatic Castration-Resistant Prostate Cancer In The United States. Value Health. 2013;16: A136. doi: 10.1016/j.jval.2013.03.664

90. Lew I. Managed care implications in castration-resistant prostate cancer. Am J Manag Care. 2013;19: S376-S381.

91. Li T, Thompson M, Todd MB, Yu MK, Kheoh TS, He JM, et al. An indirect treatment comparison (ITC) and cost-effectiveness analysis of abiraterone acetate and enzalutamide for the treatment of metastatic castration-resistant prostate cancer (mCRPC) post-chemotherapy. J Clin Oncol. 2014;32. doi: 10.1200/jco.2014.32.4_suppl.270

92. Li Y, Dehm SM. Methods for identifying and quantifying mRNA expression of androgen receptor splicing variants in prostate cancer. Methods Mol Biol. 2016;1443: 165-177. doi: 10.1007/978-1-4939-3724-0_11

93. Liepe K, Shinto A. From palliative therapy to prolongation of survival: (223)RaCl2 in the treatment of bone metastases. Ther Adv Med Oncol. 2016;8: 294-304. doi: 10.1177/1758834016640494
94. Lloyd AJ, Kerr C, Penton J, Knerer G. Health-related quality of life and health utilities in metastatic castrate-resistant prostate cancer: a survey capturing experiences from a diverse sample of UK patients. Value Health. 2015;18: 1152-1157. doi: 10.1016/j.jval.2015.08.012
95. Lorente D, Ravi P, Mehra N, Pezaro C, Omlin A, Gilman A, et al. Interrogating metastatic prostate cancer treatment switch decisions: a multi-institutional survey. Eur Urol Focus. 2016. doi: 10.1016/j.euf.2016.09.005
96. Loriot Y, Massard C, Fizazi K. Recent developments in treatments targeting castration-resistant prostate cancer bone metastases. Ann Oncol. 2012;23: 1085-1094. doi: 10.1093/annonc/mdr573
97. Macedo A, Araújo A, Melo FC, Nunes G, Cantinho G, Amorin I. [Cost-effectiveness of samarium-153-EDTMP for the treatment of pain due to multiple bone metastases in hormone-refractory prostate cancer versus conventional pain therapy, in Portugal]. Acta Med Port. 2006;19: 421-426.
98. Mahon KL, Henshall SM, Sutherland RL, Horvath LG. Pathways of chemotherapy resistance in castration-resistant prostate cancer. Endocr Relat Cancer. 2011;18: R103-R123. doi: 10.1530/erc-10-0343
99. Maia MC, Hansen AR. A comprehensive review of immunotherapies in prostate cancer. Crit Rev Oncol Hematol. 2017;113: 292-303. doi: 10.1016/j.critrevonc.2017.02.026
100. Major PP, Cook RJ. Clinical endpoints for assessing bisphosphonate efficacy in the prevention of skeletal complications of bone metasatases. European Urology Supplements. 2004;3: 34-39. doi: 10.1016/j.eursup.2004.08.010
101. Malkowicz SB. The role of diethylstilbestrol in the treatment of prostate cancer. Urology. 2001;58: 108-113. doi: 10.1016/S0090-4295(01)01252-3

102. Maran PR, Aiello A, Magri MR, Alberti C, Visentin E, Venturini F, et al. Cost effectiveness analysis in the Veneto region of cabazitaxel versus mitoxantrone in patients with metastatic hormone refractory prostate cancer, previously treated eith a docetaxel containing regimen. Value Health. 2012;15: A424. doi: 10.1016/j.jval.2012.08.2123

103. Medical Services Advisory Committee. Radium 223 for the treatment of patients with symptomatic castrate resistant prostate cancer with skeletal metastase. Canberra: Medical Services Advisory Committee; 2014.

104. Mitchell M. Comparing cost and utilization of 2 therapies for metastatic castration-resistant prostate cancer. Am Health Drug Benefits. 2015;8: 195-195.

105. Moise P, Fassler P, Holmstrom S. Lessons learned from HTA cost effectiveness evaluations of new castration-resistant prostate cancer medications. Value Health. 2012;15: A228. doi: 10.1016/j.jval.2012.03.1229

106. Moreno Gómez Á, Abajo Del Álamo C, Catalá Pindado MÁ, Godoy Díez M. [Some questions about abiraterone, breakfast and public funding]. Farm Hosp. 2015;39: 120-121. doi: 10.7399/fh.2015.39.2.8241

107. Morote J, Cozar JM, Duran I, Veiga FG, Leon L, Maroto P, et al. Cost assessment of metastatic and non-metastatic castration-resistant prostate cancer patient-management in Spain. Value Health. 2013;16: A405. doi: 10.1016/j.jval.2013.08.476

108. Moul JW, Dawson N. Quality of life associated with treatment of castration-resistant prostate cancer: a review of the literature. Cancer Invest. 2012;30: 1-12. doi: 10.3109/07357907.2011.629381

109. Moyad MA, Scholz MC. Short-term enzalutamide treatment for the potential remission of active surveillance or intermediate-risk prostate cancer: a case study, review, and the need for a clinical trial. Res Rep Urol. 2014;6: 71-77. doi: 10.2147/rru.s63136

110. Nabhan C. Sipuleucel-T immunotherapy for castration-resistant prostate cancer. N Engl J Med. 2010;363: 1966-1967; author reply 1968. doi: 10.1056/NEJMc1009982#SA2

111. Naha N, Lee HY, Jo MJ, Chung BC, Kim SH, Kim MO. Rare sugar D-allose induces programmed cell death in hormone refractory prostate cancer cells. Apoptosis. 2008;13: 1121-1134. doi: 10.1007/s10495-008-0232-7

112. Nelius T, Klatte T, de Riese W, Haynes A, Filleur S. Clinical outcome of patients with docetaxel-resistant hormone-refractory prostate cancer treated with second-line cyclophosphamide-based metronomic chemotherapy. Med Oncol. 2010;27: 363-367. doi: 10.1007/s12032-009-9218-8

113. Ngo LSM, Yeo A, Wong ASC, Tay MH. Efficacy of low-dose ketoconazole in hormone refractory prostate cancer patients at the National Cancer Centre and The Cancer Institute, Singapore. Ann Acad Med Singapore. 2007;36: 811-814.

114. Nguyen P, Je Y, Schutz F, Hoffman K, Hu J, Parekh A, et al. Association of androgen deprivation therapy with cardiovascular death in patients with prostate cancer: a meta-analysis of randomized trials. JAMA. 2011;306: 2359-2366. doi: 10.1001/jama.2011.1745

115. Nightingale G, Ryu J. Cabazitaxel (Jevtana): a novel agent for metastatic castration-resistant prostate cancer. P T. 2012;37: 440-448.

116. Norum J, Traasdahl E, Totth A, Nieder C, Olsen JA, Olsen JA. Health economics and radium-223 (Xofigo®) in the treatment of metastatic castration-resistant prostate cancer (MCRCP). A case history and a systematic review of the literature on cost-effectiveness analysis (CEA). Value Health. 2015;18: A460-A461. doi: 10.1016/j.jval.2015.09.1191

117. Obando CA, Desanvicente-Celis Z, Gonzalez L, Muschett D, Gonzalez F, Goldberg P. Cost-effectiveness analysis of abiraterone acetate treatment compared with cabacitaxel in the Republic of Panama, in patients with metastatic castration-resistant

prostate cancer that have failed to chemotherapy with docetaxel. Value Health. 2014;17: A632. doi: 10.1016/j.jval.2014.08.2261

118. Oefelein MG, Resnick MI. The impact of osteoporosis in men treated for prostate cancer. Urol Clin North Am. 2004;31: 313-319. doi: 10.1016/j.ucl.2004.02.002

119. Oellerich M, Schütz E, Beck J, Kanzow P, Plowman PN, Weiss GJ, et al. Using circulating cell-free DNA to monitor personalized cancer therapy. Crit Rev Clin Lab Sci. 2017;54: 205-218. doi: 10.1080/10408363.2017.1299683

120. Okumura H, Inoue S, Naidoo S, Holmstrom S, Akaza H. Cost-effectiveness analysis of enzalutamide for patients with chemotherapy-naive metastatic castration resistant prostate cancer in Japan. Value Health. 2017;20: A107.

121. Oudard S, Courbon F. Controversies and consensus in the innovation access for cancer therapy in the European countries: on the subject of metastatic prostate cancer. Ann Oncol. 2017;28: 421-426. doi: 10.1093/annonc/mdw546

122. Oudard S, Maroto P, Demonty G, Gerritsen WR. Charting recent progress and challenges in metastatic castration-resistant prostate cancer: is there an optimal treatment sequence? Eur Urol Focus. 2016;2: 426-440. doi: 10.1016/j.euf.2015.11.008

123. Pal SK, Stein CA, Sartor O. Enzalutamide for the treatment of prostate cancer. Expert Opin Pharmacother. 2013;14: 679-685. doi: 10.1517/14656566.2013.775251

124. Paller CJ, Antonarakis ES. Sipuleucel-T for the treatment of metastatic prostate cancer: promise and challenges. Hum Vaccin Immunother. 2012;8: 509-519. doi: 10.4161/hv.18860

125. Parente P, Parnis F, Gurney H. Challenges in the sequencing of therapies for the management of metastatic castration-resistant prostate cancer. Asia Pac J Clin Oncol. 2014;10: 205-215. doi: 10.1111/ajco.12193

126. Payne H, Bahl A, Mason M, Troup J, De Bono J. Optimizing the care of patients with advanced prostate cancer in the UK: current challenges and future opportunities. BJU Int. 2012;110: 658-667. doi: 10.1111/j.1464-410X.2011.10886.x

127. Penson DF, Lin DW, Karsh L, Quinn DI, Shevrin DH, Shore N, et al. Treatment registry for outcomes in patients with castration-resistant prostate cancer (TRUMPET): a methodology for real-world evidence and research. Future Oncol. 2016;12: 2689-2699. doi: 10.2217/fon-2016-0298

128. Peppercorn J, Armstrong A, Zaas DW, George D. Rationing in urologic oncology: lessons from sipuleucel-T for advanced prostate cancer. Urol Oncol. 2013;31: 1079-1084. doi: 10.1016/j.urolonc.2011.12.022

129. Pérez-Alcántara F, Martínez Llinàs D, Maroto JP, Gallardo E, Subirà R, Rubio M, et al. Comparative efficacy and costs of treatment sequences in metastatic castration resistant prostate cancer. Value Health. 2015;18: A458. doi: 10.1016/j.jval.2015.09.1175

130. Persson U, Nilsson S, Hjortsberg C, Prutz C. Economic evaluation of abiraterone acetate as treatment for metastatic castration resistant prostate cancer after failure of docetaxel in Sweden. Value Health. 2012;15: A219. doi: 10.1016/j.jval.2012.03.1183

131. Persson U, Nilsson S, Prutz C, Hjortsberg C. Cost effectiveness analysis of abiraterone acetate as treatment for metastatic castration resistant prostate cancer after failure of docetaxel using data from real life treatment praxis in sweden. Value Health. 2013;16: A134-A135. doi: 10.1016/j.jval.2013.03.655

132. Peskin SR. Immunotherapy for advanced prostate cancer: a novel treatment option to improve survival. Manag Care. 2011;20: 3-4, 6-11.

133. Petrylak D, Crawford E, Petrylak DP, Crawford ED. Biomarkers for the management of castration-resistant prostate cancer: we are not there yet. Target Oncol. 2017;12: 401-412. doi: 10.1007/s11523-017-0500-y

134. Pfister D, Porres D, Piper C, Thissen A, Heidenreich A. Pharmaco-economic evaluation of docetaxel-based chemotherapy in men with metastatic, castration resistant prostate cancer (mCRPC). Onkologie. 2013;36: 80-81.

135. Pham T, Sadowski MC, Li H, Richard DJ, d'Emden MC, Richard K. Advances in hormonal therapies for hormone naïve and castration-resistant prostate cancers with or without previous chemotherapy. Exp Hematol Oncol. 2015;5: 15. doi: 10.1186/s40164-016-0046-1

136. Pititto L, Asano E. Cost effectiveness of abiraterone acetate plus prednisone in the pre-chemotherapy setting followed by enzalutamide in the post-chemotherapy setting versus the opposite treatment sequence in metastatic castration-resistant prostate cancer patients under the Brazilian private health care system perspective. Value Health. 2016;19: A149. doi: 10.1016/j.jval.2016.03.1584

137. Piva M, Cordero JAC, Cabrera ES. Cost-effectiveness analysis of abiraterone in patients with metastatic, castration-resistant, prostate cancer with progression after receiving chemotherapy with docetaxel, compared with receiving only palliative support: the perspective of the Costa Rican public health system (Caja Costarricense de Seguro Social). Value Health. 2016;19: A154. doi: 10.1016/j.jval.2016.03.1615

138. Politi PM. [Thalidomide. Clinical trials in cancer]. Medicina (B Aires). 2000;60 Suppl 2: 61-65.

139. Pollard M, Moskowitz A, Oh W, Galsky M, Hall S. Cost-effective analysis (CEA) of the current treatment paradigm for metastatic castration-resistant prostate cancer (mCRPC). J Urol. 2013;189: E51-E52. doi: 10.1016/j.juro.2013.02.1507

140. Qi W, Shen Z, Yao Y. Docetaxel-based therapy with or without estramustine as first-line chemotherapy for castration-resistant prostate cancer: a meta-analysis of four randomized controlled trials. J Cancer Res Clin Oncol. 2011;137: 1785-1790.

141. Rader M, Goessl C, Cong Z. Economic evaluation of denosumab compared with zoledronic Acid in hormone-refractory prostate cancer patients with bone metastases. J Manag Care Spec Pharm. 2012;18: 74-75, author reply 75-76. doi: 10.18553/jmcp.2012.18.1.74

142. Rane JK, Pellacani D, Maitland NJ. Advanced prostate cancer--a case for adjuvant differentiation therapy. Nat Rev Urol. 2012;9: 595-602. doi: 10.1038/nrurol.2012.157

143. Reis LO. Variations of serum testosterone levels in prostate cancer patients under LH-releasing hormone therapy: an open question. Endocr Relat Cancer. 2012;19: R93-98. doi: 10.1530/erc-12-0040

144. Rentzeperi-Michalakou E, Kani C, Souliotis K, Aravantinos G, Samantas E, Ardavanis A, et al. Costs and clinical outcomes in the management of metastatic castration-resistant prostate cancer in Greece. Value Health. 2016;19: A758-A758. doi: 10.1016/j.jval.2016.09.2349

145. Renzulli JF, Collins J, Mega A. Radium-223 dichloride: illustrating the benefits of a multidisciplinary approach for patients with metastatic castration-resistant prostate cancer. J Multidiscip Healthc. 2015;8: 279-286. doi: 10.2147/jmdh.s81007

146. Rocha J, Vanhuyse M, Aprikian A, Cury F, Kassouf W, Dragomir A. Use of abiraterone in the management of castration-resistant prostate cancer: a real-life cost-effectiveness study. Value Health. 2015;18: A203-A203. doi: 10.1016/j.jval.2015.03.1174

147. Roghmann F, Antczak C, McKay RR, Choueiri T, Hu JC, Kibel AS, et al. The burden of skeletal-related events in patients with prostate cancer and bone metastasis. Urol Oncol. 2015;33: 17.e19-17.e18. doi: 10.1016/j.urolonc.2014.09.010

148. Rosenbaum SE, Wu S, Newman MA, West DP, Kuzel T, Lacouture ME. Dermatological reactions to the multitargeted tyrosine kinase inhibitor sunitinib. Support Care Cancer. 2008;16: 557-566.

149. Rothermundt C, Hayoz S, Templeton AJ, Winterhalder R, Strebel RT, Bärtschi D, et al. Metformin in chemotherapy-naive castration-resistant prostate cancer: a multicenter phase 2 trial (SAKK 08/09). Eur Urol. 2014;66: 468-474. doi: 10.1016/j.eururo.2013.12.057

150. Sartor O. State-of-the-art management for the patient with castration-resistant prostate cancer in 2012. Am Soc Clin Oncol Educ Book. 2012: 289-291. doi: 10.14694/EdBook_AM.2012.32.289

151. Sasse AP, Lopes G, Teich V, Fay AP, Abadi M, Schultz NM, et al. Number needed to treat and cost per clinically meaningful outcome of enzalutamide and abiraterone acetate for the treatment of metastatic castration-resistant prostate cancer that failed androgen deprivation therapy in Brazil. Value Health. 2016;19: A722-A722. doi: 10.1016/j.jval.2016.09.2151

152. Saylor PJ. Bone targeted therapies for the prevention of skeletal morbidity in men with prostate cancer. Asian J Androl. 2014;16: 341-347. doi: 10.4103/1008-682x.122591

153. Schmid H-P, Theiler R. Words of wisdom. Re: Economic evaluation of denosumab compared with zoledronic acid in hormone-refractory prostate cancer patients with bone metastases. Eur Urol. 2012;61: 427-428. doi: 10.1016/j.eururo.2011.11.027

154. Schmitt B, Bennett C, Seidenfeld J, Samson D, Wilt TJ. Maximal androgen blockade for advanced prostate cancer. Cochrane Database Syst Rev. 1999. doi: 10.1002/14651858.CD001526

155. Schulman CC. Intermittent hormone therapy: what is its place in clinical practice? Eur Urol Suppl. 2009;8: 852-856. doi: 10.1016/j.eursup.2009.07.005

156. Sciarra A, Abrahamsson PA, Brausi M, Galsky M, Mottet N, Sartor O, et al. Intermittent androgen-deprivation therapy in prostate cancer: a critical review focused on phase 3 trials. Eur Urol. 2013;64: 722-730. doi: 10.1016/j.eururo.2013.04.020

157. Seal B, Pawar V, Valderrama A, Grabbi E, Lloyd A, Beaudet A. Comparative analysis of radium-223 versus placebo in symptomatic metastatic castration resistant prostate cancer treated with best standard of care on skeletal-related events outcomes. Value Health. 2013;16: A133-a134. doi: 10.1016/j.jval.2013.03.650

158. Seed G, Yuan W, Mateo J, Carreira S, Bertan C, Lambros M, et al. Gene copy number estimation from targeted next generation sequencing of prostate cancer biopsies:

analytic validation and clinical qualification. Clin Cancer Res. 2017;23: 6070-6077. doi: 10.1158/1078-0432.ccr-17-0972

159. Serpa NA, Tobias-Machado M, Kaliks R, Wroclawski M, Pompeo A, Giglio A. Ten years of docetaxel-based therapies in prostate adenocarcinoma: a systematic review and meta-analysis of 2244 patients in 12 randomized clinical trials. Clin Genitourin Cancer. 2011;9: 115-123. doi: 10.1016/j.clgc.2011.05.002

160. Shah N, Dizon DS. New-generation platinum agents for solid tumors. Future Oncol. 2009;5: 33-42. doi: 10.2217/14796694.5.1.33

161. Shah-Manek B, Galanto JS, Nguyen H, Ignoffo R. Value frameworks for the patient-provider interaction: a comparison of the ASCO value framework versus NCCN evidence blocks in determining value in oncology. J Manag Care Spec Pharm. 2017;23: S13-S20. doi: 10.18553/jmcp.2017.23.6-a.s13

162. Shelley M, Harrison C, Coles B, Stafforth J, Wilt T, Mason M. Chemotherapy for hormone-refractory prostate cancer. Cochrane Database Syst Rev. 2006. doi: 10.1002/14651858.CD005247.pub2

163. Shibahara H, Shiroiwa T, Nakamura K, Shimozuma K. Cost-effectiveness analysis of abiraterone acetate as second line treatment for metastatic castration-resistant prostate cancer after docetaxel treatment in Japan. Value Health. 2013;16: A415-A416. doi: 10.1016/j.jval.2013.08.532

164. Shibahara H, Shiroiwa T, Tange C, Nakamura K, Ozono S, Shimozuma K. Reanalysis of cost-effectiveness of abiraterone ac etate as second line treatment for metastatic castration-resistant prostate cancer in Japan using a Japanese claim data set. Value Health. 2014;17: A85-A86. doi: 10.1016/j.jval.2014.03.498

165. Shinohara N, Abe T, Maruyama S. [Advancement in the treatment against prostate cancer]. Nihon Rinsho. 2016;74: 27-33.

166. Simoens S. Pharmaco-economic aspects of sipuleucel-T. Hum Vaccin Immunother. 2012;8: 506-508. doi: 10.4161/hv.18334

167. Simondsen K, Kolesar J. New treatment options for castration-resistant prostate cancer. Am J Health-Syst Pharm. 2013;70: 856-865. doi: 10.2146/ajhp110586

168. Skaltsa K, Longworth L, Ivanescu C, Phung D, Holmstrom S. Mapping the FACT-P to the preference-based EQ-5D questionnaire in metastatic castration-resistant prostate cancer. Value Health. 2014;17: 238-244. doi: 10.1016/j.jval.2013.12.005

169. Smith TJ, Dow LA, Virago EA, Khatcheressian J, Matsuyama R, Lyckholm LJ. A pilot trial of decision aids to give truthful prognostic and treatment information to chemotherapy patients with advanced cancer. J Support Oncol. 2011;9: 79-86. doi: 10.1016/j.suponc.2010.12.005

170. Snedecor SJ, Carter JA, Kaura S, Botteman M. Cost-effectiveness of zoledronic acid (ZOL) versus denosumab (Dmab) in prevention of skeletal-related events (SREs) in castration-resistant prostate cancer metastatic to the bone (mCRPC). J Clin Oncol. 2011;29. doi: 10.1200/jco.2011.29.15_suppl.4581

171. Svensson J, Andersson E, Persson U, Edekling T, Ovanfors A, Ahlgren G. Value of treatment in clinical trials versus the real world: the case of abiraterone acetate (Zytiga) for postchemotherapy metastatic castration-resistant prostate cancer patients in Sweden. Scand J Urol. 2016;50: 286-291. doi: 10.3109/21681805.2016.1172254

172. Svensson J, Andersson E, Persson U, Edekling T, Ovanfors A, Ahlgren G. Value of treatment in clinical trials versus the real world: the case of abiraterone acetate (Zytiga) for postchemotherapy metastatic castration-resistant prostate cancer patients in Sweden. Scand J Urol. 2016;50: 286-291. doi: 10.3109/21681805.2016.1172254

173. Sweeney C, Nakabayashi M, Regan M, Xie W, Hayes J, Keating N, et al. The development of intermediate clinical endpoints in cancer of the prostate (ICECaP). J Natl Cancer Inst. 2015;107: 1-8. doi: 10.1093/jnci/djv261

174. Tan SH, Bujkiewicz S, Abrams KR. Bivariate indirect comparison meta-analysis model in economic evaluation of cancer treatments. Value Health. 2013;16: A594. doi: 10.1016/j.jval.2013.08.1661

175. Tanaka G, Tsumoto K, Tsuji S, Aihara K. Bifurcation analysis on a hybrid systems model of intermittent hormonal therapy for prostate cancer. Physica D. 2008;237: 2616-2627. doi: 10.1016/j.physd.2008.03.044

176. Tannock IF, Amir E, Booth C, Niraula S, Ocana A, Seruga B, et al. Relevance of randomised controlled trials in oncology. Lancet Oncol. 2016;17: e560-e567. doi: 10.1016/S1470-2045(16)30572-1

177. Tannock IF, Osoba D, Stockler MR, Ernst DS, Neville AJ, Moore MJ, et al. Chemotherapy with mitoxantrone plus prednisone or prednisone alone for symptomatic hormone-resistant prostate cancer: A Canadian randomized trial with palliative end points. J Clin Oncol. 1996;14: 1756-1764. doi: 10.1200/JCO.1996.14.6.1756

178. Tenuta A, Klotz L, Parker JL. Clinical trial risk in castration-resistant prostate cancer: immunotherapies show promise. BJU Int. 2014;113: E82-E89. doi: 10.1111/bju.12309

179. Thompson M, Li T, Todd MB, Yu MK, Kheoh T, He JM, et al. An indirect treatment comparison and cost-effectiveness analysis of abiraterone acetate and enzalutamide for the treatment of metastatic castration-resistant prostae cancer post chemotherapy. J Urol. 2014;191: E810-E810. doi: 10.1016/j.juro.2014.02.2211

180. Todenhöfer T, Schwentner C, Schilling D, Gakis G, Stenzl A. [Treatment of metastatic bone disease and treatment-induced osteoporosis in prostate cancer. Evolution of osteoprotective strategies]. Urologe A. 2011;50: 1055-1063. doi: 10.1007/s00120-011-2623-6

181. Tsiatas M, Grivas P. Immunobiology and immunotherapy in genitourinary malignancies. Ann Transl Med. 2016;4: 270. doi: 10.21037/atm.2016.06.29

182. van Soest RJ, Efstathiou JA, Sternberg CN, Tombal B. The Natural History and Outcome Predictors of Metastatic Castration-resistant Prostate Cancer. Eur Urol Focus. 2016;2: 480-487. doi: 10.1016/j.euf.2016.12.006

183. Vasani D, Josephson DY, Carmichael C, Sartor O, Pal SK, Vasani D, et al. Recent advances in the therapy of castration-resistant prostate cancer: the price of progress. Maturitas. 2011;70: 194-196. doi: 10.1016/j.maturitas.2011.07.018

184. Vicente C, Loblaw A, North S, Kassouf W, Naidoo S, Husein F, et al. Cost-utility analysis of enzalutamide for patients with chemotherapy-naïve metastatic castration-resistant prostate cancer (MCRPC) after failure of androgen deprivation therapy (ADT). Value Health. 2015;18: A474. doi: 10.1016/j.jval.2015.09.1266

185. Virgo KS, Basch E, Loblaw DA, Oliver TK, Rumble RB, Carducci MA, et al. Second-line hormonal therapy for men with chemotherapy-naïve, castration-resistant prostate cancer: American Society of Clinical Oncology provisional clinical opinion. J Clin Oncol. 2017;35: 1952-1964. doi: 10.1200/JCO.2017.72.8030

186. Wagmiller JA, Dick AW, Sahasrabudhe DM. Cost identification of an individualized dosing strategy for lutenizing hormone releasing hormone agonist (LH-RHa) in the treatment of hormone refractory prostate cancer (HRPC). J Clin Oncol. 2004;22: S403. doi: 10.1200/jco.2004.22.90140.4588

187. Walsh PC. Re: Denosumab versus zoledronic acid for treatment of bone metastases in men with castration-resistant prostate cancer: a randomised, double-blind study. J Urol. 2011;186: 2254-2255. doi: 10.1016/j.juro.2011.08.108

188. Walsh P. Sipuleucel-T immunotherapy for castration-resistant prostate cancer: editorial comment. J Urol. 2011;185: 897-898. doi: 10.1016/j.juro.2010.11.029

189. Wen L, Valderrama A, Carlton R, Eaddy M, Seal B. Cost of treatment of radiopharmaceutical and chemotherapy for the treatment of castration-resistant prostate cancer with bone metastases in hospital setting. Value Health. 2015;18: A201. doi: 10.1016/j.jval.2015.03.1166

190. Williams S, Davis I, Sweeney C, Stockler M, Martin A, Marchesin V, et al. Randomised phase 3 trial of enzalutamide in androgen deprivation therapy with radiation therapy for

high risk, clinically localised prostate cancer: The ANZUP ENZARAD Trial (ANZUP 1303). J Clin Oncol. 2015;33. doi: 10.1200/JCO.2017.35.15_suppl.TPS5096

191. Wilson LS, Zhong L, Pon V, Srinivas S, Frear M, Nguyen N, et al. Cost effectiveness analysis of new treatments for metastatic castration-resistant prostate cancer- Does severity matter? Value Health. 2012;15: A220-A221. doi: 10.1016/j.jval.2012.03.1189

192. Wilt T, Mason M, Dahm P, Bennett C, Rutks I. Cochrane Urology Group. About The Cochrane Collaboration. 2009.

193. Wong RK, Wiffen PJ. Bisphosphonates for the relief of pain secondary to bone metastases. Cochrane Database Syst Rev. 2002. doi: 10.1002/14651858.CD002068

194. Wu EQ, Mulani P, Farrell MH, Sleep D. Mapping FACT-P and EORTC QLQ-C30 to patient health status measured by EQ-5D in metastatic hormone-refractory prostate cancer patients. Value Health. 2007;10: 408-414. doi: 10.1111/j.1524-4733.2007.00195.x

195. Yu AP, Namjoshi M, Xie J, Parikh K, Wu EQ, Guo A, et al. Economic evaluation of denosumab compared with zoledronic acid in patients with hormone-refractory prostate cancer with bone metastases. J Clin Oncol. 2011;29. doi: 10.1200/jco.2011.29.15_suppl.e15115

196. Yuen KY, Shelley M, Sze WM, Wilt T, Mason M. Bisphosphonates for advanced prostate cancer. Cochrane Database Syst Rev. 2006: N.PAG-N.PAG.
